# High species diversity of *Phintella* and *Phintella*‐like spiders (Araneae: Salticidae) in Vietnam revealed by DNA‐based species delimitation analyses

**DOI:** 10.1002/ece3.11144

**Published:** 2024-03-12

**Authors:** Luong Thi Hong Phung, Yong‐Chao Su, Takeshi Yamasaki, Yi‐Yen Li, Katsuyuki Eguchi

**Affiliations:** ^1^ Institute of Ecology and Biological Resources Vietnam Academy of Science and Technology Hanoi Vietnam; ^2^ Department of Biomedical Science and Environmental Biology Kaohsiung Medical University Kaohsiung Taiwan; ^3^ Institute of Natural and Environmental Sciences University of Hyogo Sanda Japan; ^4^ Museum of Nature and Human Activities, Hyogo University of Hyogo Sanda Japan; ^5^ Department of Biological Sciences, Graduate School of Science Tokyo Metropolitan University Hachioji Japan; ^6^ Department of International Health and Medical Anthropology Institute of Tropical Medicine, Nagasaki University Nagasaki Japan

**Keywords:** jumping spider, molecular phylogeny, RASP, sexual dimorphism, species boundaries, synonyms

## Abstract

Salticidae (jumping spiders) usually exhibit pronounced sexual dimorphism in adult morphology, particularly body coloration and size and shape of the first legs. Consequently, the male and female from the same species might be erroneously assigned to different species or even different genera, which could generate synonymies in classification if only morphological data were used. *Phintella* is a species‐rich genus of Salticidae, which currently exhibits 76 named species. However, the male–female counterpart is unknown for nearly half of the species. In this study, we used a molecular approach to delineate the species boundaries for *Phintella* and *Phintella*‐like specimens collected in Vietnam, using morphological information as supporting data. We used three gene fragments (mitochondrial COI, 16S‐ND1, and nuclear 28S) and biogeographical considerations for species delimitation. A total of 22 putative species were recognized: 18 species of the genus *Phintella*, one species of the genus *Lechia* (*L. squamata*), and three species of the genus *Phinteloides*. Eleven undescribed species were discovered, of which seven have a male–female combination, two species have only males, and two species have only females. The crown age of *Phintella* was estimated at the Serravallian stage of the Miocene after the increase of species number around 16 MYA. The crown ages of most putative species recognized in this study were estimated in the Pleistocene, and the divergence among sister species likely occurred from the mid‐Miocene to the Pliocene. Our ancestral range reconstruction results showed that the diversification of our ingroup was governed by progressive dispersal events, i.e., *Phintella* and their related species in Vietnam diversified while expanding their range on the continent. Our results provide fundamental biodiversity data for a high‐diversity genus in Vietnamese *Phintella* spiders.

## Graphical abstract

We documented 22 putative species of jumping spiders in the genus Phintella and its closely related taxa that exhibit distinct sexual dimorphism in adult morphology. Our phylogenetic analyses and species delimitation analyses showed that there are 13 undescribed species. This species‐rich taxon was estimated diversified at the Serravallian, Miocene, followed by a high diversification rate in the Pleistocene.

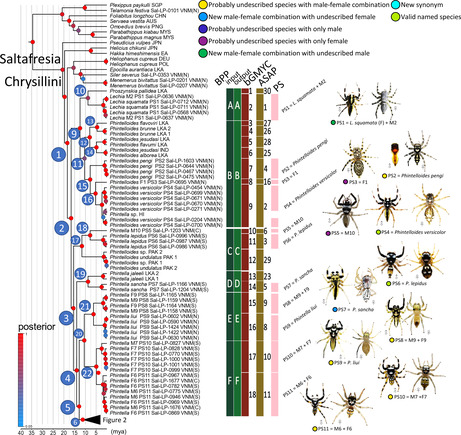

## 1 INTRODUCTION

The order Araneae (spiders) is a major group of predatory arthropods in terrestrial ecosystems (Foelix, 1996). The global spider community consumes 400–800 million tons of prey annually, approximately 0.1% of the global terrestrial net primary production (Nyffeler & Birkhofer, 2017). Salticidae, the most species‐rich spider family (Bodner & Maddison, 2012; Richman, 1982), consists of 6640 described species and 681 genera, holding 12.8% of all species of Araneae (51,917 species, World Spider Catalog, updated February 15, 2024). In spiders, the morphology of male and female copulatory organs (male palp and female epigyne) has features related to mechanical reproductive isolation, which have been intensively used in delineating the boundaries among species or higher taxa (Foelix, 1996). However, the current classification of Salticidae, especially the records in Southeast Asia, is primarily based on morphology, which may involve many errors and obscurities in taxon recognition (e.g., Phung et al., 2016). As Salticidae usually exhibit distinct sexual dimorphism in adult morphology, particularly body coloration, body size, and shape of the first legs, conspecific males and females might often be erroneously assigned to different species or genera. Given that genetics or behaviors are not available for matching conspecific males and females, many synonymies might be hidden in the current classification (Logunov & Jaeger, 2015; Phung et al., 2016; Żabka, 1985).

Species delimitation using a combination of molecular phylogenetic analyses and morphological examination has been widely applied to various animal taxa (Derkarabetian & Hedin, 2014; Diaz et al., 2015; Gonzalez et al., 2014; Kergoat et al., 2015; Montagna et al., 2016; Su et al., 2016). Arachnologists are vigorously elucidating the species diversity of spiders and updating the conventional classification using molecular data while taking morphological identification into consideration (Ballarin & Eguchi, 2023; Li et al., 2022; Lo et al., 2021; Macharoenboon et al., 2021). Such approaches are practical in solving the taxonomic problems and obscurities in the species recognition and classification of Salticidae, which are primarily due to remarkable sexual dimorphism in adult morphologies (Bopearachchi et al., 2022; Kanesharatnam & Benjamin, 2021; Phung et al., 2016). Moreover, Maddison et al. (2020) demonstrated the effectiveness of a genomic approach to draw species boundaries in Salticidae with an example of the tribe Baviini, whereas the genus‐level definition and species‐level recognition are still primarily based on morphology (Prószyński, 2016a, 2016b, 2019).

The jumping spiders in the genus *Phintella* are no exception to the taxonomic obscurities originating from their strong sexual dimorphism. *Phintella* is a species‐rich genus in Salticidae, and 76 named species with cosmopolitan distribution are included in this genus (World Spider Catalog, 2024). The male–female combination is unknown for about 1/3 number of the species, i.e., 15 species have male‐only specimens, and 10 species have female‐only specimens. For example, Żabka (1985) described *Phintella aequipeiformis* Żabka, 1985, based on a male, and *P. lucai* Żabka, 1985, was based on a female in his monograph of Vietnamese Salticidae. Since then, the opposite sex of each nominal species has remained unknown. Phung et al. (2016) revealed the conspecificity of the two named species based on the results of DNA barcoding and field collection data and synonymized them under the name *P. aequipeiformis*.

As a case study to shed light on two major problems in our precise understanding of the local species diversity of jumping spiders, i.e., the presence of cryptic species as well as taxonomic confusion caused by strong sexual dimorphism, our present study aims to conduct the species‐level delimitation of *Phintella* and *Phintella*‐like species intensively collected from Vietnam using molecular‐based species delimitation using three gene fragments (mitochondrial COI, 16S‐ND1, and nuclear 28S) and biogeographical considerations. We used different algorithms to delineate the species boundaries using molecular data. Then, we identified the opposite sex for the putative species for which only one sex has been known. In addition, the divergence time and the diversification rate shift of the biogeographic histories of the major lineages and putative species were also estimated to reveal the governing processes of diversification in *Phintella* and their phylogenetically related species.

## 2 MATERIALS AND METHODS

### 2.1 Target taxa

**FIGURE 1:**
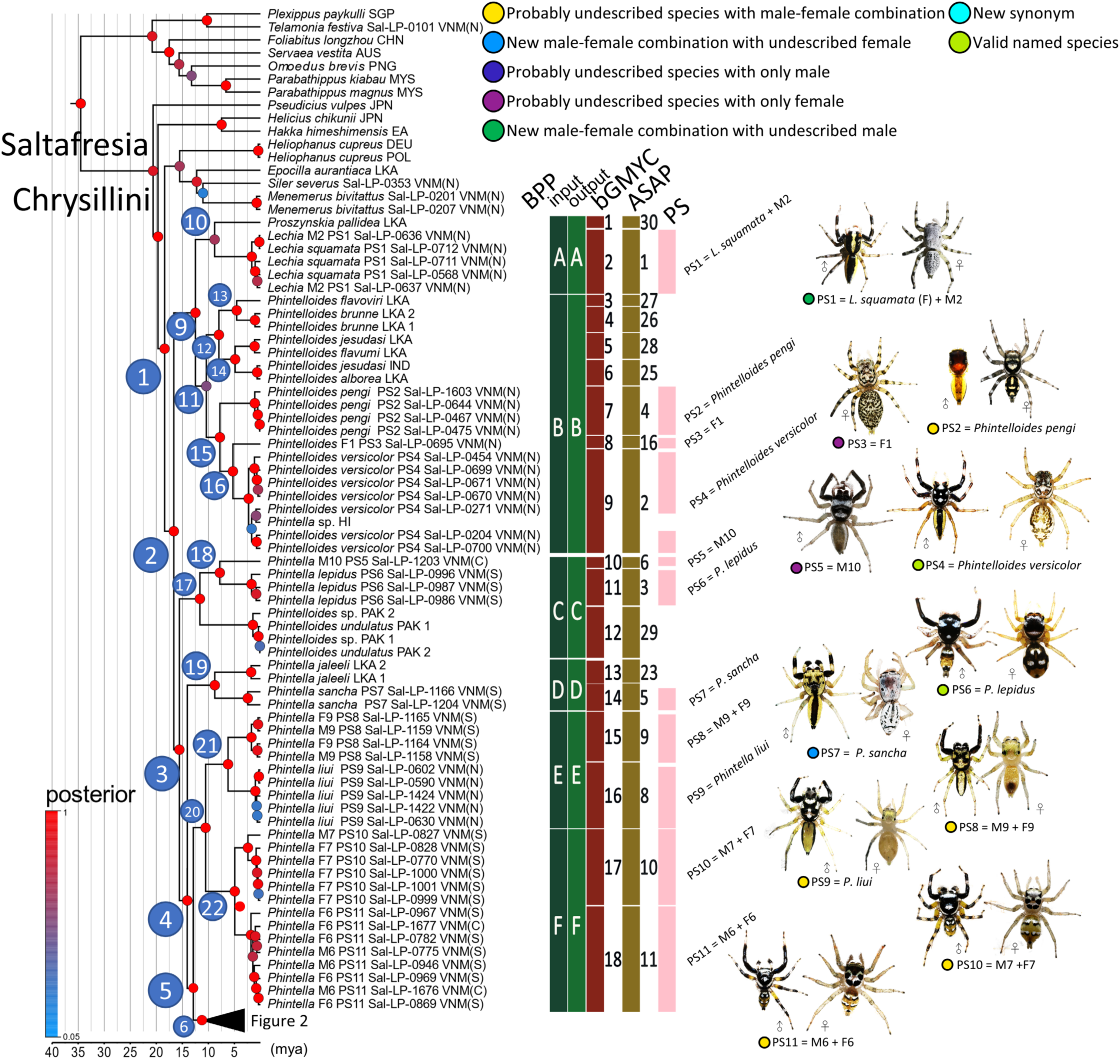
Maximum credibility phylogenetic tree (MCC tree) reconstructed using BEAST and the summary of species delimitation results (part I). The MCC tree reconstructed via BEAST showed that the 22 morphospecies (the PS column in pink) corresponded to monophyletic clades, indicating they are potentially valid species. The species delimitation results of BFD, bGMYC, and ASAP supported the species boundaries of the morphospecies in different degrees. The result of ASAP supported a 30‐species scenario, and the result of bGMYC supported a 37‐species scenario. For more conservative methods, BFD supported a nine‐species scenario, and bGMYC supported a 11‐species scenario.

**FIGURE 2:**
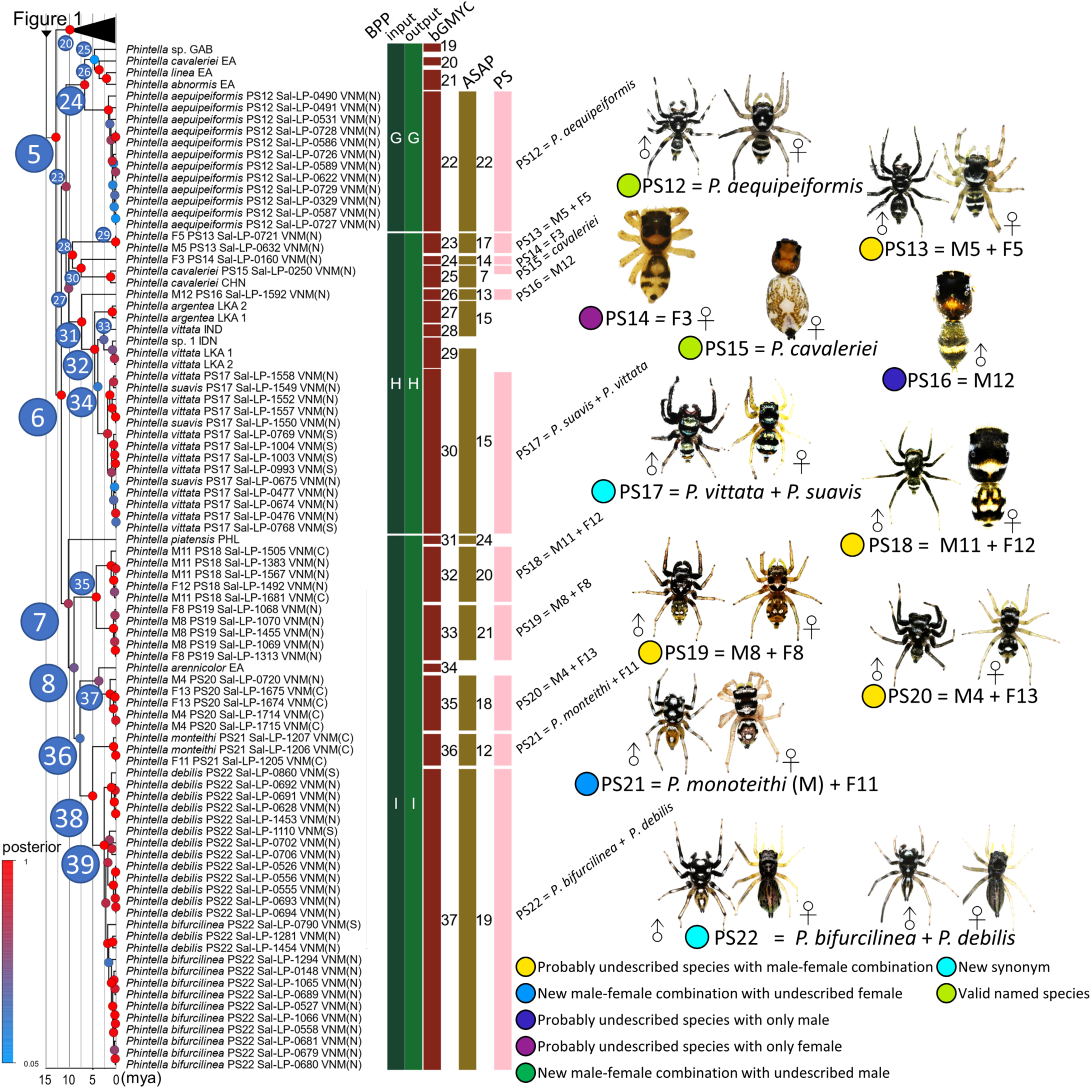
Maximum credibility phylogenetic tree (MCC tree) reconstructed using BEAST and the summary of species delimitation results (part II, continued Figure 1).

Vietnamese species and populations of the genus *Phintella* and their closely related species are the targets of this study because the diversification of this genus has not been closely examined. In the current classification of Salticidae (World Spider Catalog, 2024), the following eight valid named species of *Phintella* have been recorded from Vietnam (Kanesharatnam & Benjamin, 2019; Peng & Xie, 1995; Żabka, 1985, 2012): *P. accentifera* (Simon, 1901); *P. aequipeiformis* Żabka, 1985; *P. argenteola* (Simon, 1903); *P. bifurcilinea* (Bösenberg et Strand, 1906); *P. debilis* (Thorell, 1891); *P. daklak* (Hoang et al., 2023); *P. suavis* (Simon, 1885); and *P. vittata* (Koch, 1846). A total of 121 *Phintella* and *Phintella*‐like adult specimens collected in 19 localities of Vietnam, with an additional 43 DNA sequences (32 specimens) from Kanesharatnam and Benjamin (2019), were included as ingroups in our molecular phylogenetic analyses (Table S1; Figures 1 and 2). The 121 newly collected specimens were preliminarily assigned to 33 morphospecies, including 13 described species, 10 undescribed male morphospecies, and 10 undescribed female morphospecies. Prior to the molecular phylogenetic analyses, we preliminarily assigned the specimens into male‐based and female‐based morphospecies and made efforts to identify them by referring to previous taxonomic studies (Bösenberg & Strand, 1906; Cao et al., 2016; Kanesharatnam & Benjamin, 2019; Koch, 1846; Phung et al., 2016; Prószyński, 1984, 2016a, 2016b, 2019; Schenkel, 1963; Simon, 1885; Thorell, 1891; Wang et al., 2023; Wang & Li, 2021; Żabka, 1985, 2012) and by directly observing the following types of materials.

*Phintella accentifera* (Simon, 1901). Syntypes (11 males, 22 females, 7 juveniles); locality: Kodaikanal, India; depository: Muséum national d'Histoire naturelle (MNHN) 10,254.
*Phintella argenteola* (Simon, 1903). Holotype (male); Nghe An province, Vietnam; MNHN 22226.
*Phintella aequipeiformis* Żabka, 1985. Holotype (male); Lao Cai province, Vietnam; Hungarian Natural History Museum (HNHM).
*Phintella lucai* Żabka, 1985. Holotype (female); Yen Bai province, Vietnam; HNHM.
*Phintella suavis* (Simon, 1885). Holotype (male); Malacca, Malaysia; MNHM 4172.
*Phintella vittata* (Koch, 1846). Holotype (female); Bintang, Indonesia; Museum für Naturkunde Berlin (ZMB 1746).

A total of 16 specimens belonging to 14 species of eight genera of Salticidae were used as outgroups (Table S1). Three of these species, *Menemerus bivitattus* (Dufour, 1831), *Siler severus* (Simon, 1901), and *Telamonia festiva* (Thorell, 1887), were collected from Vietnam in this study. All sequences obtained in this study were deposited in GenBank (Table S1).

### 2.2 DNA extraction, polymerase chain reaction (PCR) amplification, and sequencing

We conducted DNA extraction, PCR amplification, and Sanger sequencing following the method of Su and Smith, 2014 and Phung et al. (2016). We sequenced three gene regions: COI (495–1413 bp of the mitochondrial cytochrome oxidase I), 16S‐ND1 (501–907 bp region from the middle of ribosomal RNA 16S to the middle of NADH dehydrogenase subunit I, with an intervening tRNA^LEU(CUN)^), and 28S (341–1094 bp of the nuclear‐encoded large subunit (28S) ribosomal repeat). The COI region is commonly used for species delimitation in various animal taxa (Hebert et al., 2003; Kekkonen & Hebert, 2014; Pires & Marinoni, 2010), and the 16S‐ND1 and 28S regions are commonly used for molecular phylogenetic analyses in Salticidae (Bodner & Maddison, 2012; Hedin & Maddison, 2001; Maddison & Hedin, 2003; Maddison et al., 2007, 2008, 2014). Sequence information of the primer sets is provided in Table 1. The PCR conditions (following Su & Smith, 2014) to amplify COI, 16S‐ND1, and 28S were set as follows: initial denaturation at 94°C for 2 min; 35 cycles of 10 s at 98°C, 30 s at either 52°C (COI) or 44°C (16S‐ND1) or 55°C (28S), and 55 s (COI) or 45 s (16S‐ND1) or 50 s (28S) at 68°C; and a final cycle of 7 min at 68°C. The forward and reverse strands were assembled after trimming the primer regions and disarrayed parts. The ambiguous sites were manually confirmed by referring to the chromatograms of the two strands using ChromasPro 2.1.5 (Technelysium Pty Ltd., Australia).

**TABLE 1:** Primers are used for amplification and sequencing.

Gene	Primer name	Primer sequence	Reference
COI	C1‐J‐1718 (F)	5′ GGAGGATTTGGAAATTGATTAGTTCC 3′	Simon et al. (1994)
	C1‐LP‐F‐1718 (F)	5′ GGAGG**T**TTTGGAAATTGATTAGTTCC 3′	Simon et al. (1994) (Modified from the original: A to T at the 6th bp (as highlighted in bold gothic))
	C1‐LP‐R‐2667 (R)	5′ CCAGCYATAATAGCAAATACAGCYC 3′	This study
	C1‐N‐2776 (R)	5′ GGATAATCAGAATATCGTCGAGG 3′	Hedin and Maddison (2001)
	C1‐N‐2797 (R)	5′ GGTAATCTGAATAACGTCGAGG 3′	Simon et al. (1994) (reverse primer of C1‐J‐2797)
16S‐ND1	16S‐ND1‐WPM‐F1 (F)	5′ GCRTCTCTRAAAGGTTG 3′	Zhang and Maddison (2013)
	16S‐ND1‐WPM‐R2 (R)	5′ GTGCTAAGGTAGCATAATA 3′	Zhang and Maddison (2013)
	16S‐ND1‐WPM‐R3 (R)	5′ CGCCTGTTTAACAAAAAC 3′	Zhang and Maddison (2013)
28S	28S‐TY‐F3 (F)	5′ ATGTGGCGTTTAGGAGTGAGC 3′	Yamasaki, unpubl.
	28S‐13‐R (R)	5′ GCT ATC CTG AGG GAA ACT TCG G 3′	van der Auwera et al. (1994)
	28S‐LP‐R1 (R)	5′ CCACCAGAGTTTCCTCTGGCTT 3′	This study

Abbreviations: F, forward primer; R, reverse primer.

### 2.3 Sequence dataset preparation

We align the sequences using the Geneious aligner algorithm in Geneious prime 2022.2 (https://www.geneious.com) by setting parameters as default (Alignment type = Global alignment with free end gaps, Cost Matrix = 65% similarity (5.0/−4.1), Gap Open Penalty = 12, Gap Extension Penalty = 3, refinement iterations = 2) and automatically detect direction. We prepared two sequence alignments for phylogenetic analyses, species delimitation analyses, and divergence time estimation: a concatenated dataset of the mitochondrial gene regions COI and 16S‐ND1 (COI + 16S‐ND1 dataset; 2414 bp) and one nuclear gene 28S (1217 bp). In addition, a COI dataset (trimmed to 1414 bp, 143 OTUs) was constructed, including COI sequences of *Phintella* species registered in GenBank and newly sequenced in this study (Table S1). Such sequences were used for Assemble Species by Automatic Partitioning (ASAP) analysis to test the species delimitation hypothesis based on the two previous datasets (Table S2).

### 2.4 Phylogenetic analyses and divergence time estimation

Using the concatenated dataset, we used the Bayesian inference to reconstruct the maximum clade credibility (MCC) tree in BEAST 1.10.4 (Drummond et al., 2012). We determined each nucleotide substitution model for each gene region (COI, 16S‐ND1, and 28S) in jModelTest (Posada, 2008). The input files for BEAST were generated by BEAUti 1.10.4 (Drummond et al., 2012). We set the nucleotide substitution model of each gene. We set the substitution rates and clock models of each gene as unlinked, the clock model as an uncorrelated relaxed clock with gamma‐relaxed distribution, and a speciation model as the Yule Process Tree Model. We chose Yule Process because we followed the protocol from Bodner and Maddison (2012). Other priors of the parameters were set as default, whereas the molecular clock of the time calibration scheme was set as follows: the MCMC chain length was 1.2 × 10^8^ with a sampling frequency of 1 × 10^4^. We visualized the results of independent runs in Tracer 1.7.1 (Rambaut et al., 2018) to diagnose the convergence of independent runs and examine the sufficiency of effective sample sizes (ESS > 200). We performed a burned‐in of 40% of trees to generate the final MCC tree using TreeAnnotator 1.10.4 (Drummond et al., 2012), which was displayed by using Figtree version 1.4.4 (Rambaut, 2018).

The divergence times were estimated through Bayesian analysis using BEAST 1.10.4, based on the concatenated datasets, with three‐time constraints. Following Bodner and Maddison (2012), we assigned 35.2 million years ago (MYA) for the node age of the most recent common ancestor (MRCA) of the clade Saltafresia of the subfamily Salticinae (prior setting: normal distribution, mean = 35.2, SD = 1.0). We assigned 18.6 MYA for the node age of the MRCA of *Phintella*, *Heliophanus*, and *Menemerus* (prior setting: normal distribution, mean = 18.6, SD = 1.0). Following Brower (1994), we applied a commonly used arthropod mitochondrial COI molecular clock, with 2.3% divergence per MY (prior setting: lognormal distribution, mean = 0.0115, SD = 0.1). We used these three‐time calibrations to estimate the divergence time of the phylogenetic tree.

### 2.5 Species delimitation analyses using molecular data

We used three approaches for species delimitation, including one barcode gene‐based method, ASAP (Puillandre et al., 2020), one Bayesian implementation of the General Mixed Yule Coalescent analysis (bGMYC, Reid & Carstens, 2012), and one Bayesian coalescent‐based method, Bayesian Phylogenetics and Phylogeography (BPP, Yang, 2015). We used the concatenated dataset for BPP and bGMYC analyses, and only the COI matrix was used for ASAP.

The bGMYC is the Bayesian version of GMYC (Pons et al., 2006). The GMYC uses only one ultrametric gene tree as an input and searches the threshold at which branching patterns represent coalescent or speciation events (Emmanuel et al., 2015; Pons et al., 2006). The bGMYC provides the means to use the posterior distribution of multiple ultrametric trees instead of a single tree (Emmanuel et al., 2015; Reid & Carstens, 2012). The 1201 trees obtained from post‐burn‐in trees generated from BEAST analysis using LogCombiner 1.10.4 (Drummond et al., 2012) were used to perform bGMYC analyses. The bGMYC was conducted in the R program using the package “bGMYC.” We ran bGMYC under each of the 1000 tree topologies, with 5 × 10^4^ MCMC generations, burn‐in of 4 × 10^4^, and thinning per 100 generations. ASAP analysis applies a hierarchical clustering algorithm that only uses pairwise genetic distances for building partitions from a DNA sequence dataset of a group of individuals. We performed ASAP at the web interface (https://bioinfo.mnhn.fr/abi/public/asap/) by selecting the JC69 model for the COI dataset. We set the default parameters in “Advanced Option.” The partition with the lowest “ASAP score” was selected as the best under a given substitution model.

We used the Bayesian coalescent‐based method, BPP (Yang, 2015), to delimitate species boundaries based on the BEAST tree topology. The species status was determined based on the monophyly of the clades under similar morphology and geographic distribution. Accordingly, nine monophyletic groups were set as putative species, and the species of each monophyletic clade was estimated using BPP v 4.4.0 (Flouri et al., 2018). We assumed a guide tree topology from the BEAST tree results and then applied the gamma prior for theta and root tau (gamma 2 1000). BPP analysis was processed using an MCMC chain of 2 × 10^5^ after discarding the first 8000 steps.

### 2.6 Diversification rate shift and ancestral range reconstruction

Based on the species delimitation results, we prepared a final MCC tree from BEAST analysis of the proposed species. We first kept one tip but dropped the other tips in a putative species (species estimated via ASAP and bGMYC as the most specious estimation) and kept the closely related outgroup in the tree. Then, diversification rate shift estimation was conducted using BAMM‐2.5.0 (Rabosky, 2014) with four chains, 10 million generations, and simulatePriorShifts = 0. Priors were set as the recommendation of BAMMtools (Rabosky et al., 2014) for the final MCC tree. Finally, visualized the results by R package BAMMtools (Rabosky et al., 2014) to identify the rate shifts in the MCC tree generated via BEAST using the lineage‐through‐time (LTT) approach (Nee et al., 1994). LTT estimations were conducted using the R package ape (v5.7.1, Paradis & Schliep, 2019) for the MCC tree and 1000 randomly selected trees from BEAST analysis, then all LTT plots and evolutionary rates visualized by BAMMtools were incorporated by using ggplot2 (Wickham, 2011). The effective sample size (ESS) was evaluated by R package CODA (Plummer et al., 2006); furthermore, functions from BAMMtools were used in the analysis below: we determined the expected number of shifts by computeBayesFactors() and summary. bammdata(), and then the credible set of diversification rate shift configurations were estimated by credibleShiftSet() with the expected number of shifts set to 0. Finally, we visualized the speciation rate by plot. bamm(). The ancestral ranges of each node in the ingroup were also reconstructed using the currently known distribution of the putative species (Table S1). We conducted ancestral range model selection to identify the best statistics for reconstruction. The possible models are DIVALIKE, DEC, and BAYAREALIKE implemented in BioGeoBEARs with a *j*‐parameter representing the long‐distance dispersal scenario (Matzke, 2013a, 2013b, 2014). When the best model was identified, ancestral range reconstruction was conducted under BioGeoBEARS methods in Reconstruct Ancestral State in Phylogenies (RASP V.4, Yu et al., 2015). The geographic divisions used in the analysis were based on Bain and Hurley (2011). We define our geographic areas in the RASP analysis according to the number of species included in our analyses and the approximate geographic areas. We divided Vietnam into Northern, Central, and Southern Vietnam. For the areas outside of Vietnam, we designate India, Pakistan, and Sri Lanka as one area; and the Philippines and Indonesia as one area. We set Hawaii, East Asia, and Africa, respectively, as one area.

## 3 RESULTS

### 3.1 BEAST tree reconstruction and species delimitations

Our reconstructed MCC tree (Figures 1 and 2) showed that the 33 *Phintella* and *Phintella*‐like morphospecies form a monophyletic group, i.e., the clade after node 2 (hereafter Clade 2, posterior probability (PP) = 1.00). The same applies to other nodes, which are grouped into two major clades. The first clade (Clade 9, PP = 0.99, Figure 1) contains *Phintelloides versicolor*, F1, *P. pengi*, *P. jesudasi*, *P. flavoriri*, *P. alborea*, *P. flavumi*, *P. brunne*, *Proszynskia pallidea*, and *Lechia squamata* + M2. The second clade (Clade 3; PP = 1.00, Figure 1) contains all *Phintella* species. Clade 17 (PP = 1.00), which consists of *Phintelloides undulatus*, *Phintelloides* sp. PAK2, *P. lepidus*, and M10 are the first clade diverged in Clade 3. Afterward, clade 19 (PP = 1.00), which consists of *P. sancha* and *P. jaleeli*, is separated from Clade 4. Clade 4 (PP = 1.00) involves several highly supported inner clades consisting of multiple morphospecies. Clade 21 (PP = 1.00) contains “M9 + F9,” which is sister to “*P. liui*”. Clade 22 (PP = 1.00) contains “M7 + F7,” which is sister to “M6 + F6” (Figure 1).

As shown in Figure 2, Clade 24 (PP = 0.94) contains *P. aequipeiformis*, which is sister to the low‐support Clade 25 (PP = 0.05) of *P. abnormis* EA, *P. linea* EA, *P. cavaleriei* EA, and *P*. sp. GAB (EA = East Asia, GAB = Gabon, Africa, and see other region codes in Table S3). Clade 28 (PP = 1.00) contains “M5 + F5,” which is sister to Clade 30 (PP = 1.00) of *P. cavaleriei* and F3. Clade 31 (PP = 0.98) contains M12, which is sister to Clade 32 (PP = 0.98) of “*P. vittata* + *P. suavis*” and *P. argentea* LKA. Clade 35 (PP = 1.00) contains “M11 + F12,” which is sister to “M8 + F8.” Clade 37 (moderate support, PP = 0.56) contains “M4 + F13,” which is sister to *P. arennicolor* EA. Clade 38 (PP = 1.00) contains “*P. debilis* + *P. bifurcilinea*” (Clade 39; PP = 1.00), which is sister to “*P. monteithi* + F11.”

The following number of putative species were recovered in the ingroups by species delimitation analyses (the number of putative species from Vietnam was given in round parentheses): 30 (22) in ASAP, 37 (22) in bGMYC, and nine (9) in BFD. Species delimitation in BFD was much more conservative than that in ASAP and bGMYC (Figures 1 and 2).

### 3.2 Divergence time and diversification rate shift estimations

**FIGURE 3:**
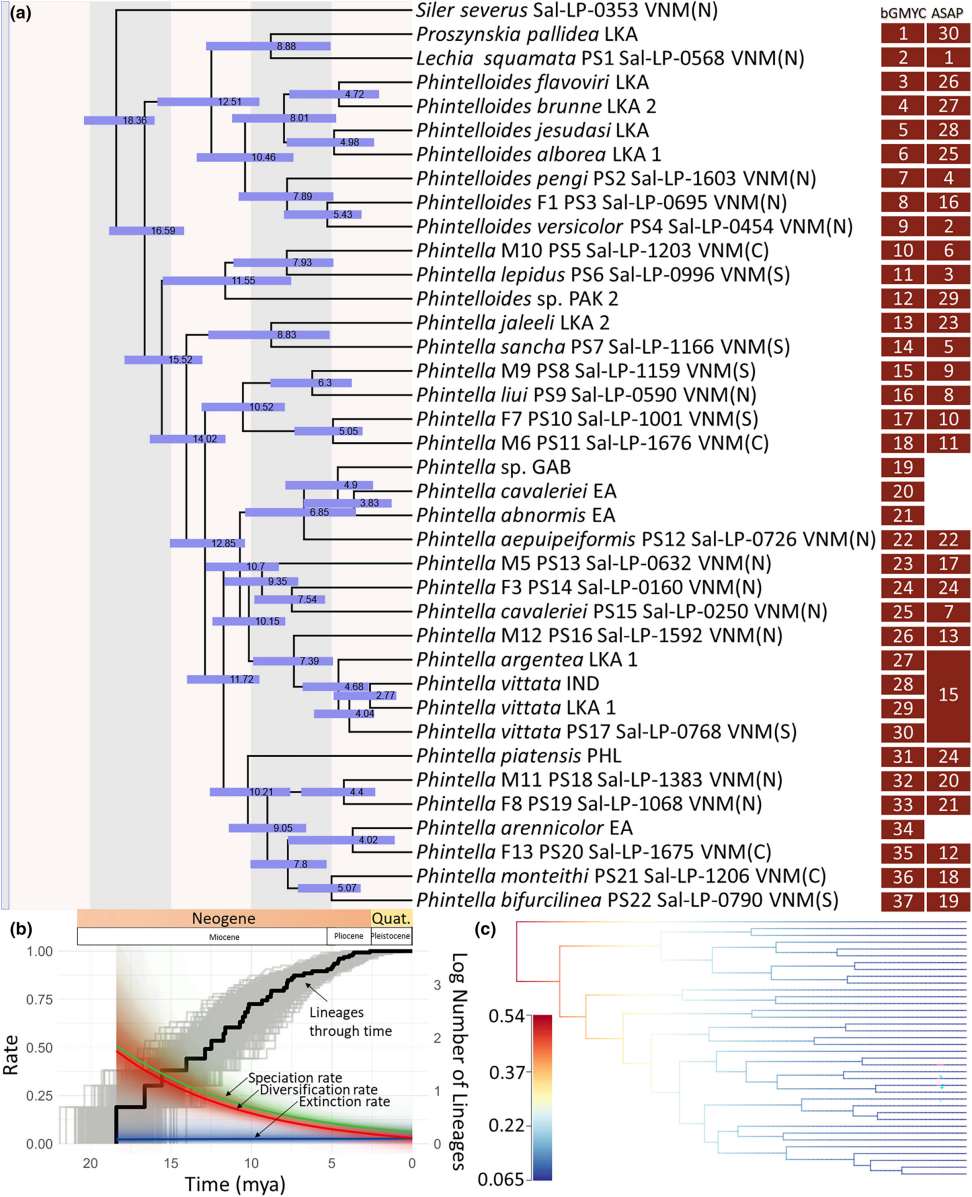
Divergence time estimations and diversification rate under the lineage through time approach using the species delimitated via ASAP. (a) The divergence time of the Phintella and the ingroup started at 18.36 MYA. The major clades in Vietnam started diverging after 15.52 MYA. (b) The diversification rate, speciation rate, and extinction rate are demonstrated in the lineage through time plot, the gray lines represent another 1000 LTT plots from BEAST analysis, and shades of each color represent confidence of evolutionary rate calculated by BAMMtools, i.e., the speciation events were not changed through the geological time from Pliocene to Pleistocene (5.00–2.50 MYA). (c) The speciation rate was calculated via BAMM. There is no shift in the best diversification rate shift configuration (posterior probability = 0.8).

By using the MCC tree from BEAST analyses and keeping one tip per putative species (37 species scenarios in bGMYC), we show the estimated divergence time of each clade and their inferred diversification rate for a given period, which we did not detect a significant rate shift in our ingroup; rather, the diversification rate is higher during mid‐Miocene, ~15–16 MYA, then slowly decline (Figure 3b). Our time estimation of Clade 2 shows that the two major clades split in 16.59 MYA (95% highest probability density (HPD) = 14.18–18.84 MYA). The crown age of the *Phintella* clade (Clade 3) and its sister Clade 9 was estimated to be 15.52 MYA (HPD = 13.04–17.89 MYA) and 12.51 MYA (HPD = 9.51–15.84 MYA), respectively. Accordingly, the major divergence in our ingroup taxa occurred in the mid‐Miocene (Figure 3a,b). The LTT plot shows that the cladogenesis of *Phintella* and related lineages in Vietnam proceeded with an increase of lineage from the middle to late Miocene (12.00–7.50 Ma), which slowed down and flattened throughout late Miocene and Pliocene (7.50–2.50 Ma), and to Pleistocene (2.50 Ma to current, Figure 3b,c). Other diversification rate shift configurations have very low posterior probabilities (Figure S1), and thus were not considered in our study.

### 3.3 Ancestral range and biogeographic process reconstructions

**FIGURE 4:**
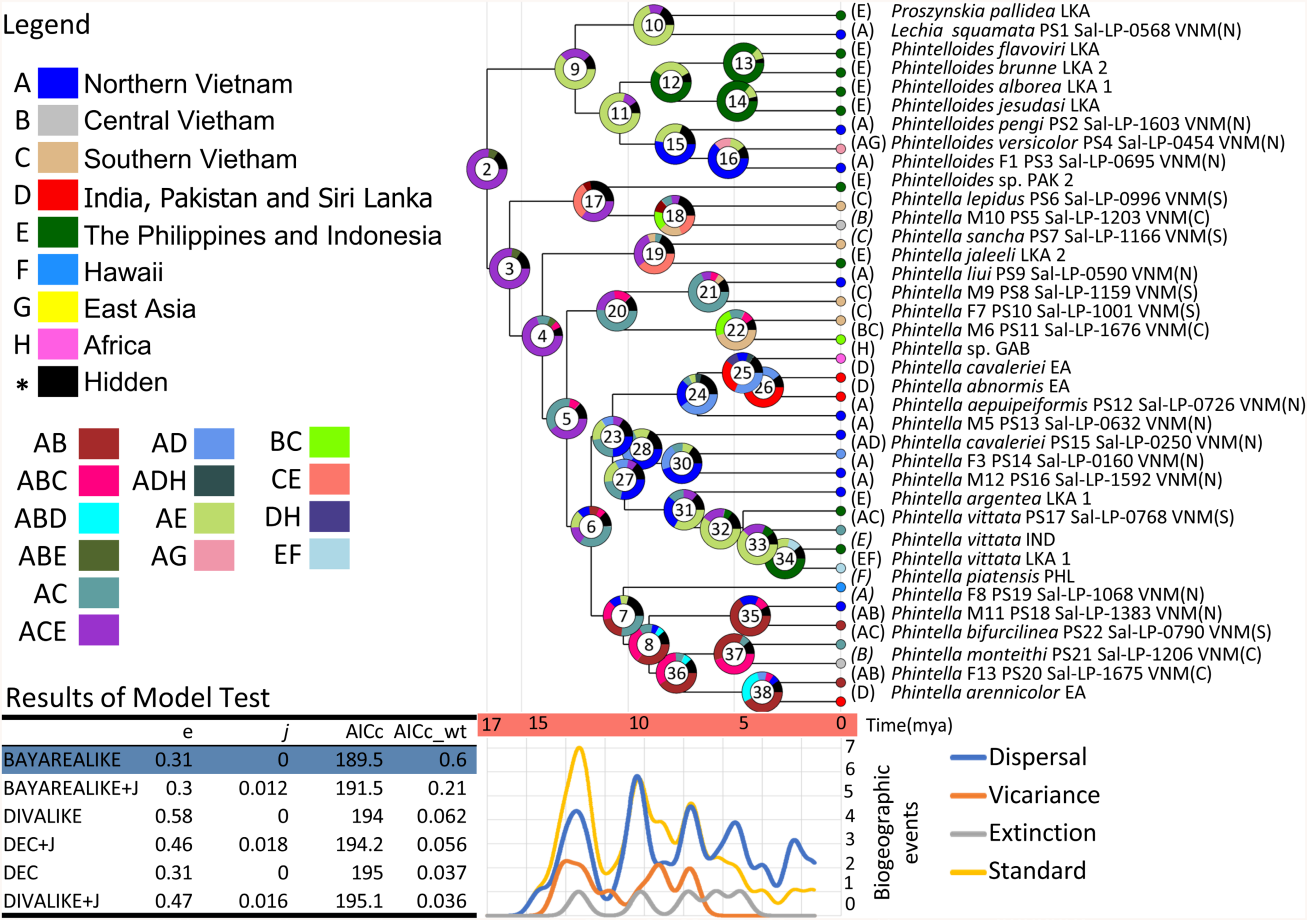
The ancestral range reconstructions and the estimation of historical biogeographic events. The RASP analyses show that the best explanatory model is the DEC model (AICc wt = 0.60). The ancestral range reconstruction results under the DEC model show that long‐distance dispersal (the *J*‐parameter = 0) did not govern the diversification of the species, instead, short‐range dispersal was controlling the species diversification in our target taxa.

RASP analyses demonstrated that the best explanatory biogeographic model for our ingroup is the BAYAREALIKE model (AICc_wt = 0.60, Figure 4) under the scheme of Northern Vietnam (area A), Central Vietnam (area B), Southern Vietnam (area C), India + Pakistan + Sri Lanka (area D), the Philippines + Indonesia (area E), Hawaii (area F), East Asia (area G), and Africa (area H) in ancestral range reconstructions. The *J*‐parameter represents that the count of long‐distance dispersal event (*J* = 0) is insignificant in this model. Based on the reconstructed ancestral range, the ancestral range of Clade 2 is likely Northern Vietnam (area A) + Southern Vietnam (C) + the Philippines + Indonesia (area E); (thus ACE for Clade 2 = 76.69%, Figure 4). The ancestral range of the majority of the SE Asian *Phintella* species (Vietnam, Sri Lanka, India, and Pakistan) is likely to be ACE (Clade 3 = 77.36%, Clade 4 = 70.46%, Clade 5 = 40.33%). This result could be biased because the species are collected from Vietnam. However, the reconstructed ancestral area indicated a SE Asian continental origin (and its neighboring island systems) scenario of the putative species recognized in this study.

By contrast, the “non‐*Phintella*” clade (Clade 9) has been primarily diversified in Northern Vietnam and the Philippines + Indonesia (AE). Moreover, the plot of biogeographic events showed that the governing biogeographic process for the diversification of our ingroup species was dispersal. This result indicated species diversification was due to dispersal and expansion to the adjacent geographic ranges because the long‐distance dispersal parameter (*J*) is insignificant.

## 4 DISCUSSION

### 4.1 Species delimitation hypothesis and taxonomic remarks

The BFD species delimitation hypothesis with nine species is overly conservative and unreasonable because it proposes the merging of multiple morphological species that can be distinguished based on important characteristics found in somatic and genital morphology. Apart from the BFD hypothesis, 22 ingroup putative species (PS1–PS22) collected from Vietnam are recognized by considering the agreement between ASAP and bGMYC delimitation (Figures 1 and 2). A total of 11 species are considered to be undescribed species. Of these, the conspecific male–female combination was elucidated for seven species (PS8, PS10, PS11, PS13, PS18, PS19, and PS20), while the only male was found for two species (PS5 and PS16), and the only female was found for two species (PS3 and PS14). Our divergence time estimation showed that the major divergence occurred in the middle to late Miocene but with our significant diversification rate shift. Pleistocene events have little effect on species diversification in our ingroup species (Figure 3). The biogeographic process that increases the number of species through time in our ingroup was the range expansion events via dispersing and diversification to the adjacent ranges in SE Asian landmasses (Figure 4).

The ingroup species were assigned to Clade 3 and Clade 9. Clade 3 consists of 18 putative species from Vietnam, including eight named species and two cases of the merging of named species, namely, PS22 (*Phintella bifurcilinea* + *P. debilis*), PS21 (*P. monteithi*), PS12 (*P. aequipeiformis*), PS17 (*P. vittata* + *P. suavis*), PS15 (*P. cavaleriei*), PS9 (*P. liui*), PS7 (*P. sancha*), and PS6 (*P. lepidus*); the remaining 10 species in Clade 3 are likely new species (Figures 1 and 2). Considering that *P. bifurcilinea* is the type species of the genus *Phintella*, the members of Clade 3 can be reasonably assigned to *Phintella*. The male and female specimens of *Phintella bifurcilinea* (Bösenberg & Strand, 1906) and those of *P. debilis* (Thorell, 1891) were partly mixed to form a single clade (PS22) with a high node support and a relatively long basal branch in ASAP and bGMYC. In addition, the male and female specimens of *Phintella vittata* and the male specimens of *P. suavis* were recovered as a single species (PS17). *Phintella vittata* (Koch, 1846) and *P. suavis* (Simon, 1885) were described based on different sexes, i.e., the former based on the female holotype from Bintang (Java), Indonesia, and the latter based on the male holotype from Malacca, Malaysia, thereby hindering us to discuss the conspecificity based on the direct comparison of the two species. Therefore, accepting the DNA‐based merging of the two named species is reasonable. In Clade 9, multiple species (including PS2, PS3, and PS4) of Clade 11 were morphologically assigned to *Phintelloides*. However, *Phintelloides undulatus* (Caleb & Karthikeyani, 2015) is sister to Clade 17, consisting of *Phintella lepidus* (PS6) and PS5, and is nested in *Phintella* (Clade 3). Therefore, the concept of *Phintelloides* must be further studied based on a more comprehensive taxon sampling.

Among the 22 putative species in the ingroup taxa, we identified the opposite sex of two species: *Phintella monteithi* Żabka, 2012 (male‐based species), and *Lechia squamata* Żabka, 1985 (female‐based species) were reasonably inferred based on molecular phylogenetic analyses (Figure 1). Candidate new species were also recognized from Vietnam (Figures 1 and 2). PS8 and PS9 are sister allopatric species, which are collected from Chu Yang Sin National Park and Vu Quang National Park, respectively. PS10 and PS11 are partly and allopatrically distributed sister species as they co‐occurred in the Phu Quoc National Park. The same pattern was observed in PS18 and PS19, where both species co‐occurred in Pu Mat (Table 2). Although we have newly recorded the morphology of males and females in nine species, we additionally recorded four species in that we only collected one sex. PS3, which is closely related to *P. versicolor*, and PS14, which is closely related to *P. cavaleriei*, have only one female specimen in each species. PS5, which is closely related to *P. lepidus*, and PS16, which is closely related to *P. vittata*, have only one male specimen in our collection. PS20 was recovered as the sister species of *P. arennicolor*.

**TABLE 2:** The newly collected putative species or the newly collected sex of a known species in our study.

Species label	Scientific name	Collection site(s)	N of female	N of male	Note
Named species but only one sex was previously collected but the opposite sex collected in this study					
PS21	*Phintella monteithi*	Yok Don National Park	*N* = 1 (F11, new)	*N* = 2	Following Żabka (2012)
PS7	*Phintella sancha*	Chu Yang Sin National Park	*N* = 1 (new, F10)	*N* = 1	Following Cao et al. (2016)
PS1	*Lechia squamata*	Na Hang Nature Reserve Vu Quang National Park	*N* = 1	*N* = 2 (new, M2)	Following Żabka (1985)
PS2	*Phintelloides pengi*	Me Linh Biodiv. Stat.	*N* = 3 (new, F2)	*N* = 1 (new, M1)	
PS9	*Phintella liui*	Vu Quang National Park Ben En National Park	*N* = 2 (new, F4)	*N* = 3 (new, M3)	Allopatric sister species with PS8
Undescribed species with both sexes collected					
PS8	Undescribed species	Chu Yang Sin National Park	*N* = 2 (new, F9)	*N* = 2(new, M9)	Allopatric sister species with PS9
PS10	Undescribed species	Lo Go Xa Mat National Park, K'Bang district Phu Quoc National Park	*N* = 5 (new, F7)	*N* = 1 (new, M7)	Partly allopatric sister species
PS11	Undescribed species	Phu Quoc National Park	*N* = 5 (new, F6)	*N* = 3 (new, M6)	
PS13	Undescribed species	Vu Quang National Park	*N* = 1 (new, F5)	*N* = 1 (new, M5)	
PS18	Undescribed species	Ba Vi National Park Ben En National Park Pu Mat National Park	*N* = 1 (new, F12)	*N* = 4 (new, M11)	Partly allopatric sister species
PS19	Undescribed species	Xuan Lien Pu Mat National Park Dakrong K'Bang	*N* = 2 (new, F8)	*N* = 3 (new, M8)	
PS20	Undescribed species	K'Bang Vu Quang National Park	*N* = 2 (new, F13)	*N* = 3 (new, M4)	
Species of only one sex collected					
PS16	Undescribed species	Ben En Ntional Park	NA	*N* = 1 (new, M12)	Closely related to *P. vittata*
PS14	Undescribed species	Tam Dao National Park	*N* = 1 (new, F3)	NA	Closely related to *P. cavaleriei*
PS3	Undescribed species	Na Hang National Park	*N* = 1 (new, F1)	NA	Closely related to *P. versicolor*

*Note*: We collected nine species of sexually dimorphic *Phintella* and *Phintella*‐like species that are either undescribed in both sexes, and three species with only one sex were recorded in the previous studies. *N*, denotes the number of specimens; NA denotes “not available”; new denotes newly discovered species or sex in a known species. The same color shaded the sister species pair. Two male specimens of Phintella monteithi and a single undescribed female of F11 were collected from the same location in Yok Don National Park. They are merged as the same species (PS21). A single male specimen of *P. sancha* and a single undescribed female of F10 were collected from the same location in Chu Yang Sin National Park, which were merged as the PS7. Three female specimens of *Lechia squamata* and two undescribed male specimens of M2, collected from Na Hang Nature Reserve (one female) and Vu Quang National Park (both male and female), are merged as PS1. The formal descriptions of newly found sexes will be conducted in a separate taxonomy work. PS8 (M9 + F9, collected from Chu Yang Sin National Park) and PS9 (M3 + F4, collected from Vu Quang National Park and Ben En National Park) were recovered as two species (Figure 1). These sister clades, PS8 and PS9, are allopatric (Figure 4) thus their heterospecificity is suggested. PS10 (M7 + F7, collected from Lo Go Xa Mat National Park, K'Bang district, and Phu Quoc National Park) and PS11 (M6 + F6, exclusively in Phu Quoc National Park) were recovered as two species in ASAP and bGMYC. The sympatric occurrence of these sister lineages may suggest a presence of reproductive isolation. PS18 (M11 + F12, Ba Vi, Ben En, and Pu Mat) and PS19 (M8 + F8, Xuan Lien, Pu Mat, Dakrong, and K'Bang) were recovered as two species. These two clades are partly sympatric and are morphologically well distinguished (e.g., body coloration shown in Figure 1). Therefore, it is quite likely that PS19 and PS18 are heterospecific. PS16 (M12) was supported as an independent species in our results, and is distinguishable well in male's morphology from its phylogenetically close *P. vittata* (e.g., body color shown in Figure 1). Therefore, it is concluded that PS16 and *P. vittata* are heterospecific. PS14 (F3) was supported as an independent species in ASAP analysis, but it was combined with *P. cavaleriei* in bGMYC. However, PS14 (female) is distinguishable well in female's morphology from its sister *P. cavaleriei* (e.g., body color shown in Figure 1). Therefore, it is likely that PS14 and *P. cavaleriei* are heterospecific. PS3 (F1) was supported as an independent species in ASAP analysis and bGMYC, and is distinguishable well in female's morphology from its sister *P. versicolor* (e.g., body color shown in Figure 1). Therefore, it is likely that PS3 and *P. versicolor* are heterospecific.

### 4.2 Phylogeography and biogeography

The age of Saltafresia (Figure 1), estimated as 34.5 Mya (95% HPD = 32.54–36.44 MYA), is slightly younger than that (35.2 MYA) reported by Maddison (2015). On the contrary, the age of Chrysillini (Figure 1), or the clade included in our ingroup and its closely related clades (*Heliophanus*, *Helicius*, *Pseudicius*, *Hakka*, *Epocilla*, *Menemerus*, *and Siler*), obtained from our analyses is 18.36 MYA (95% HPD = 16.02–20.40 MYA), which is slightly older than that (18.6 MYA) reported by Maddison (2015). However, the age differences between our analyses and those reported by Maddison (2015) are negligible. In both cases, Saltafresia was dated in the early Eocene, and Chrysillini was dated in the early Miocene. The split of Clade 3 and 9 likely occurred in the mid‐Miocene (16.59 MYA, HPD = 14.18–18.84 MYA HPD). The crown age of the genus in Clade 3 was dated around the Serravallian stage to the Langhian stage of Miocene (15.52 MYA, 13.04–17.89 MYA).

The cladogenesis of *Phintella* (Clade 3) and its sister lineage (Clade 9) in Vietnam became a relatively stable increase of species (but no diversification rate shift) from the middle to late Miocene (~16.00–7.50 Ma); however, there is no increase of species throughout the early Pleistocene (2.50 Ma) (summarized in Figure 3 and Figure S1). The former phase corresponds to the period after the Middle Miocene Climate Transition, a global drastic change from a warm/wet phase to a cold/dry phase (Methner et al., 2020). This climate change likely modified the spatial distribution of vegetation types (Wang et al., 2019) and their linkages and temporally formed several suitable habitat patches temporally during the transitional period (such as open/sparse forests, forest edges, and bushes). No long‐distance dispersal was inferred for the present dataset (i.e., *J‐* parameter = 0), which indicated that dispersal across different landmasses may not always be the case. Rather, regional dispersal and vicariance events could occur at relatively small geographic scales, although jumping spiders could conduct “ballooning” (Jiménez‐Valverde et al., 2010).

### 4.3 Prediction of the species richness of *Phintella* and *Phintella*‐like spiders in Vietnam

Eight species of the genus *Phintella* were previously recorded from Vietnam (Żabka, 1985, Żabka, 2012, Peng and Xie, 1995, Kanesharatnam & Benjamin, 2019, Hoang et al., 2023). In the present study, 18 species of *Phintella* (Clade 3) were found. However, *Phintella accentifera*, *P. argenteola*, and *P. daklak* previously recorded from Vietnam were not discovered in the present study. In addition, 10 putative species (*P. monteithi*, *P. cavaleriei*, *P. sancha*, PS3, 5, 8, 10, 16, 13, and 14) were restricted to a narrow geographic range. These facts indicate that *Phintella* and *Phintella*‐like spiders' species diversity in Vietnam may still be underestimated. The putative species recovered by multiple species delimitation analyses based on the dataset of the three genes (COI, 16S, and 28S) was recovered well by ASAP analysis based on the COI dataset alone (Figures 1 and 2). Similarly, COI‐based DNA barcoding (or “mini‐barcoding” targeting a shorter COI gene marker: Meusnier et al., 2008; Vamos et al., 2017) can also be useful to preliminarily elucidate undescribed species and unknown conspecific male–female pairs in *Phintella* and its related genera. Apart from the Vietnamese species, additional species other than our targeted geographic areas, for example, China, the Philippines, and other Southeast Asian countries, should be included in future studies to illustrate the evolution and diversification histories of *Phintella* species.

## AUTHOR CONTRIBUTIONS

**Luong Thi Hong Phung:** Conceptualization (equal); data curation (equal); funding acquisition (equal); methodology (equal); resources (equal); writing – original draft (equal); writing – review and editing (equal). **Yong‐Chao Su:** Data curation (equal); formal analysis (equal); funding acquisition (equal); methodology (equal); resources (equal); supervision (equal); visualization (equal); writing – review and editing (equal). **Takeshi Yamasaki:** Data curation (supporting); investigation (supporting); methodology (supporting); resources (supporting); writing – review and editing (equal). **Yi‐Yen Li:** Formal analysis (equal); visualization (equal). **Katsuyuki Eguchi:** Conceptualization (equal); funding acquisition (equal); methodology (equal); project administration (equal); resources (equal); supervision (equal); writing – review and editing (equal).

## CONFLICT OF INTEREST STATEMENT

We have no conflict of interest.

### OPEN RESEARCH BADGES

This article has earned an Open Data badge for making publicly available the digitally‐shareable data necessary to reproduce the reported results. The data is available at https://doi.org/10.5061/dryad.9p8cz8wmz.

## Supporting information


Figure S1

Table S1

Table S2

Table S3

## Data Availability

DNA sequences: Genbank accessions LC105655–LC105663, LC105665–LC105672, OP178652–OP178891, OP204510–OP204520, and OP204522–OP204618. DNA alignments, sequence matrices, and trees from the analyses: https://doi.org/10.5061/dryad.9p8cz8wmz.
