# Supplementary material for: High species diversity of Phintella and Phintella‐like spiders (Araneae: Salticidae) in Vietnam revealed by DNA‐based species delimitation analyses
Source: Ecol Evol. 2024 Mar 12;14(3):e11144. doi: 10.1002/ece3.11144 (PMC10932738; doi:10.1002/ece3.11144)
Supplement: Supplementary file 1 — Figure S1 [file ECE3-14-e11144-s001.docx]

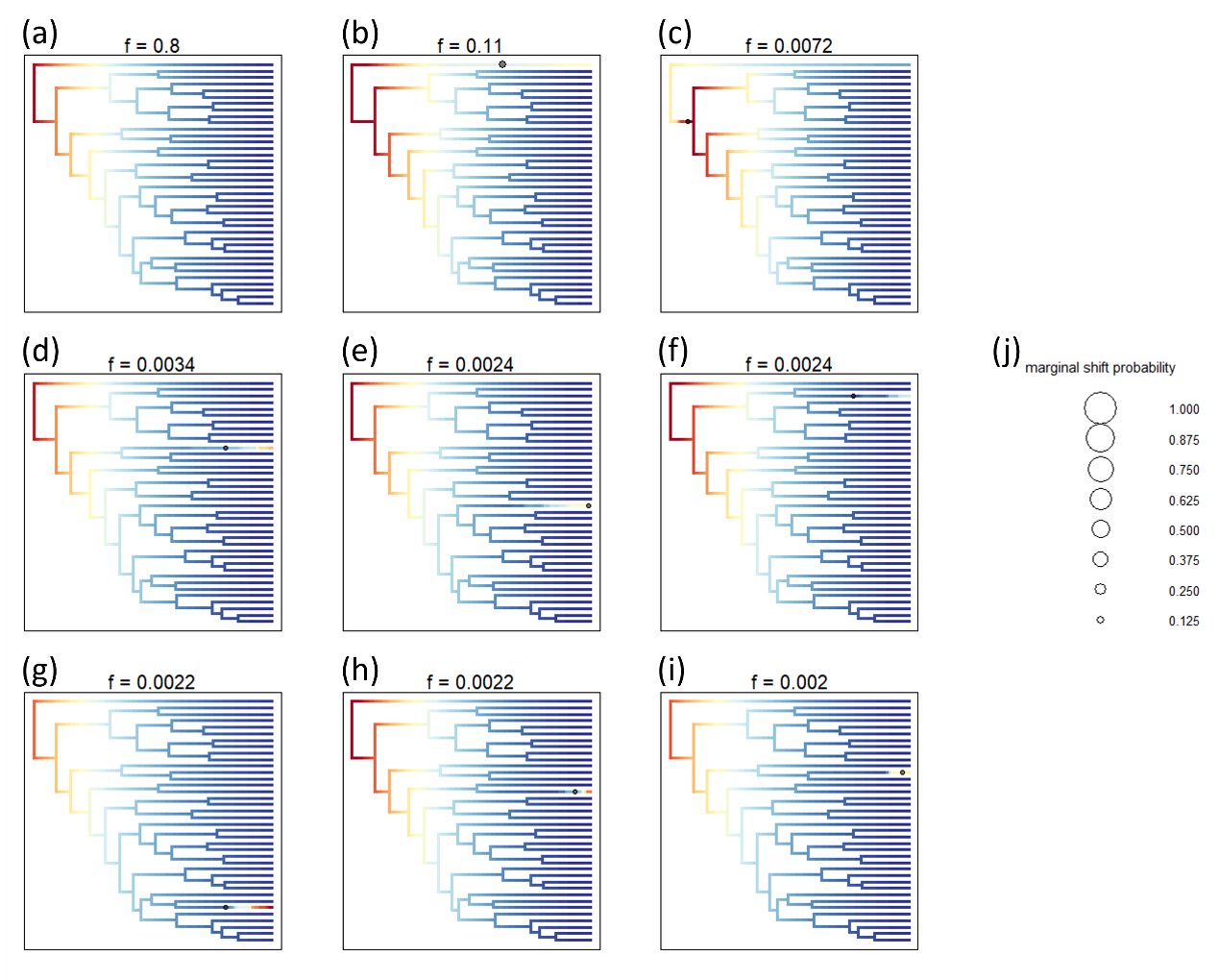


Figure S1. The 95% Credible set of shift configurations from BAMM analysis.

The nine distinct shift configurations are ordered with their corresponding posterior probabilities from high to low (all configurations encompass 93.18% of the posterior distribution). (a) The best (primary) shift configuration, with a posterior probability of 80%, the number of shifts is zero. (b) to (i) show similar configurations but with very low posterior probabilities.
