# Supplementary material for: High species diversity of Phintella and Phintella‐like spiders (Araneae: Salticidae) in Vietnam revealed by DNA‐based species delimitation analyses
Source: Ecol Evol. 2024 Mar 12;14(3):e11144. doi: 10.1002/ece3.11144 (PMC10932738; doi:10.1002/ece3.11144)
Supplement: Supplementary file 2 — Table S1 [file ECE3-14-e11144-s003.docx]

Table S1. Specimens examined in the present study.

The number in round parentheses after “Locality” are corresponded to those in Figure 1 and Table 1.

Abbreviations: *P.*, *Phintella*; PS, putative species; M, male-based morphospecies; F, female-based morphospecies; NP, National Park; NR, Nature Reserve.

| **Morphospecies** | **ID code** | **PS** | **Locality abbre** | **Locality** | **Sex** | **Lat.** | **Lon.** | **COI** | **16S-ND1** | **28S** |
| --- | --- | --- | --- | --- | --- | --- | --- | --- | --- | --- |
| *Heliophanus cupreus* | n/a |  | POL | Poland | n/a |  |  | DQ665756.1 | KM032916.1 | EF201653.1 |
| *Foliabitus longzhou* | n/a |  | CHN | China | n/a |  |  | KC615757.1 | KC615999.1 | KC615577.1 |
| *Omoedus brevis* | n/a |  | PNG | Papua New Guinea | n/a |  |  | KC615791.1 | KC616043.1 | KC615620.1 |
| *Parabathippus kiabau* | n/a |  | MYS | Malaysia | n/a |  |  | KC615657.1 | KC615833.1 | KC615405.1 |
| *Parabathippus magnus* | n/a |  | MYS | Malaysia | n/a |  |  | KC615656.1 | KC615830.1 | KC615402.1 |
| *Plexippus paykulli* | n/a |  | SGP | Singapore | n/a |  |  | JX145699.1 | JX145921.1 | JX145784.1 |
| *Servaea vestita* | n/a |  | AUS | Australia | n/a |  |  | KC615769.1 | KC616014.1 | KC615592.1 |
| *Telamonia festiva* | Sal-LP-0101 |  | VNM(N) | Me Linh Biodiv. Stat. | M | 21.384 | 105.712 | OP204618.1 | OP178891.1 | OP178771.1 |
| *Phintella bifurcilinea* | Sal-LP-0148 | PS22 | VNM(N) | Me Linh Biodiv. Stat. | M | 21.384 | 105.712 | LC105655.1 | OP178792.1 | OP178672.1 |
| *Phintella* F3 | Sal-LP-0160 | PS14 | VNM(N) | Tam Dao NP | F | 21.464 | 105.646 | OP204510.1 | OP178780.1 | OP178660.1 |
| *Menemerus bivitattus* | Sal-LP-0201 |  | VNM(N) | Bach Long Vi I. | M | 20.132 | 107.731 | OP204615.1 | OP178888.1 | OP178768.1 |
| *Phintelloides versicolor* | Sal-LP-0204 | PS4 | VNM(N) | Bach Long Vi I. | M | 20.132 | 107.731 | LC105656.1 | OP178870.1 | OP178750.1 |
| *Menemerus bivitattus* | Sal-LP-0207 |  | VNM(N) | Bach Long Vi I. | F | 20.132 | 107.731 | OP204616.1 | OP178889.1 | OP178769.1 |
| *Phintella cavaleriei* | Sal-LP-0250 | PS15 | VNM(N) | Sa Pa | F | 22.336 | 103.834 | OP204511.1 | OP178781.1 | OP178661.1 |
| *Phintelloides versicolor* | Sal-LP-0271 | PS4 | VNM(N) | Sa Pa | M | 22.336 | 103.834 | LC105657.1 |  |  |
| *Phintella aepuipeiformis* | Sal-LP-0329 | PS12 | VNM(N) | Xuan Son NP | M | 21.124 | 104.973 | LC105658.1 | OP178772.1 | OP178652.1 |
| *Siler severus* | Sal-LP-0353 |  | VNM(N) | Sa Pa | M | 22.336 | 103.834 | OP204617.1 | OP178890.1 | OP178770.1 |
| *Phintelloides versicolor* | Sal-LP-0454 | PS4 | VNM(N) | Dong Thai, Ba Vi, Hanoi | F | 21.226 | 105.398 | OP204596.1 | OP178867.1 | OP178747.1 |
| *Phintelloides* *pengi* | Sal-LP-0467 | PS2 | VNM(N) | Me Linh Biodiv. Stat. | M | 21.384 | 105.712 | OP204602.1 | OP178875.1 | OP178755.1 |
| *Phintelloides* *pengi* | Sal-LP-0475 | PS2 | VNM(N) | Me Linh Biodiv. Stat. | F | 21.384 | 105.712 | OP204604.1 | OP178877.1 | OP178757.1 |
| *Phintella vittata* | Sal-LP-0476 | PS17 | VNM(N) | Dong Mo | M | 21.033 | 105.469 | OP204546.1 | OP178817.1 | OP178697.1 |
| *Phintella vittata* | Sal-LP-0477 | PS17 | VNM(N) | Dong Mo | F | 21.033 | 105.469 | OP204547.1 | OP178818.1 | OP178698.1 |
| *Phintella aepuipeiformis* | Sal-LP-0490 | PS12 | VNM(N) | Na Hang NR | F | 22.471 | 105.309 | LC105659.1 | OP178773.1 | OP178653.1 |
| *Phintella aepuipeiformis* | Sal-LP-0491 | PS12 | VNM(N) | Na Hang NR | M | 22.471 | 105.309 | LC105660.1 | OP178774.1 | OP178654.1 |
| *Phintella debilis* | Sal-LP-0526 | PS22 | VNM(N) | Na Hang NR | M | 22.471 | 105.309 | OP204526.1 | OP178797.1 | OP178677.1 |
| *Phintella bifurcilinea* | Sal-LP-0527 | PS22 | VNM(N) | Na Hang NR | M | 22.471 | 105.309 | OP204523.1 | OP178794.1 | OP178674.1 |
| *Phintella aepuipeiformis* | Sal-LP-0531 | PS12 | VNM(N) | Na Hang NR | M | 22.471 | 105.309 | LC105661.1 | OP178779.1 | OP178659.1 |
| *Phintella debilis* | Sal-LP-0555 | PS22 | VNM(N) | Na Hang NR | M | 22.471 | 105.309 | OP204524.1 | OP178795.1 | OP178675.1 |
| *Phintella debilis* | Sal-LP-0556 | PS22 | VNM(N) | Na Hang NR | F | 22.471 | 105.309 | OP204527.1 | OP178798.1 | OP178678.1 |
| *Phintella bifurcilinea* | Sal-LP-0558 | PS22 | VNM(N) | Na Hang NR | M | 22.471 | 105.309 | OP204522.1 | OP178793.1 | OP178673.1 |
| *Lechia squamata* | Sal-LP-0568 | PS1 | VNM(N) | Na Hang NR | F | 22.471 | 105.309 | OP204606.1 | OP178879.1 | OP178759.1 |
| *Phintella aequipeiformis* | Sal-LP-0586 | PS12 | VNM(N) | Vu Quang NP | M | 18.286 | 105.477 | LC105662.1 |  |  |
| *Phintella aequipeiformis* | Sal-LP-0587 | PS12 | VNM(N) | Vu Quang NP | M | 18.286 | 105.477 | LC105663.1 |  |  |
| *Phintella aepuipeiformis* | Sal-LP-0589 | PS12 | VNM(N) | Vu Quang NP | F | 18.286 | 105.477 | LC105665.1 | OP178776.1 | OP178656.1 |
| *Phintella* *liui* | Sal-LP-0590 | PS9 | VNM(N) | Vu Quang NP | M | 18.286 | 105.477 | OP204575.1 | OP178846.1 | OP178726.1 |
| *Phintella* *liui* | Sal-LP-0602 | PS9 | VNM(N) | Vu Quang NP | F | 18.286 | 105.477 | OP204576.1 | OP178847.1 | OP178727.1 |
| *Phintella aepuipeiformis* | Sal-LP-0622 | PS12 | VNM(N) | Vu Quang NP | M | 18.286 | 105.477 | LC105666.1 | OP178775.1 | OP178655.1 |
| *Phintella debilis* | Sal-LP-0628 | PS22 | VNM(N) | Vu Quang NP | F | 18.286 | 105.477 | OP204529.1 | OP178800.1 | OP178680.1 |
| *Phintella* *liui* | Sal-LP-0630 | PS9 | VNM(N) | Vu Quang NP | M | 18.286 | 105.477 | OP204577.1 | OP178848.1 | OP178728.1 |
| *Phintella* M5 | Sal-LP-0632 | PS13 | VNM(N) | Vu Quang NP | M | 18.286 | 105.477 | OP204512.1 | OP178782.1 | OP178662.1 |
| *Lechia* *squamata* M2 | Sal-LP-0636 | PS1 | VNM(N) | Vu Quang NP | M | 18.286 | 105.477 | OP204608.1 | OP178881.1 | OP178761.1 |
| *Lechia* *squamata* M2 | Sal-LP-0637 | PS1 | VNM(N) | Vu Quang NP | M | 18.286 | 105.477 | OP204609.1 | OP178882.1 | OP178762.1 |
| *Phintelloides* *pengi* | Sal-LP-0644 | PS2 | VNM(N) | Vu Quang NP | F | 18.286 | 105.477 | OP204601.1 | OP178874.1 | OP178754.1 |
| *Phintelloides versicolor* | Sal-LP-0670 | PS4 | VNM(N) | Dong Thai, Ba Vi, Hanoi | F | 21.226 | 105.398 | LC105667.1 | OP178871.1 | OP178751.1 |
| *Phintelloides versicolor* | Sal-LP-0671 | PS4 | VNM(N) | Dong Thai, Ba Vì, Hanoi | M | 21.226 | 105.398 | OP204599.1 | OP178872.1 | OP178752.1 |
| *Phintella vittata* | Sal-LP-0674 | PS17 | VNM(N) | Me Linh Biodiv. Stat. | F | 21.384 | 105.712 | OP204548.1 | OP178819.1 | OP178699.1 |
| *Phintella suavis* | Sal-LP-0675 | PS17 | VNM(N) | Me Linh Biodiv. Stat. | M | 21.384 | 105.712 | OP204549.1 | OP178820.1 | OP178700.1 |
| *Phintella bifurcilinea* | Sal-LP-0679 | PS22 | VNM(N) | Me Linh Biodiv. Stat. | M | 21.384 | 105.712 | OP204515.1 | OP178785.1 | OP178665.1 |
| *Phintella bifurcilinea* | Sal-LP-0680 | PS22 | VNM(N) | Me Linh Biodiv. Stat. | F | 21.384 | 105.712 | OP204516.1 | OP178786.1 | OP178666.1 |
| *Phintella bifurcilinea* | Sal-LP-0681 | PS22 | VNM(N) | Me Linh Biodiv. Stat. | F | 21.384 | 105.712 | LC105668.1 | OP178787.1 | OP178667.1 |
| *Phintella bifurcilinea* | Sal-LP-0689 | PS22 | VNM(N) | Dong Mo | F | 21.033 | 105.469 | OP204517.1 | OP178788.1 | OP178668.1 |
| *Phintella debilis* | Sal-LP-0691 | PS22 | VNM(N) | Na Hang NR | M | 22.471 | 105.309 | OP204533.1 | OP178804.1 | OP178684.1 |
| *Phintella debilis* | Sal-LP-0692 | PS22 | VNM(N) | Na Hang NR | M | 22.471 | 105.309 | OP204534.1 | OP178805.1 | OP178685.1 |
| *Phintella debilis* | Sal-LP-0693 | PS22 | VNM(N) | Na Hang NR | F | 22.471 | 105.309 | OP204536.1 | OP178807.1 | OP178687.1 |
| *Phintella debilis* | Sal-LP-0694 | PS22 | VNM(N) | Na Hang NR | F | 22.471 | 105.309 | OP204538.1 | OP178809.1 | OP178689.1 |
| *Phintelloides* F1 | Sal-LP-0695 | PS3 | VNM(N) | Na Hang NR | F | 22.471 | 105.309 | OP204600.1 | OP178873.1 | OP178753.1 |
| *Phintelloides versicolor* | Sal-LP-0699 | PS4 | VNM(N) | Vu Quang NP | M | 18.286 | 105.477 | OP204598.1 | OP178869.1 | OP178749.1 |
| *Phintelloides versicolor* | Sal-LP-0700 | PS4 | VNM(N) | Vu Quang NP | F | 18.286 | 105.477 | OP204597.1 | OP178868.1 | OP178748.1 |
| *Phintella debilis* | Sal-LP-0702 | PS22 | VNM(N) | Vu Quang NP | F | 18.286 | 105.477 | OP204535.1 | OP178806.1 | OP178686.1 |
| *Phintella debilis* | Sal-LP-0706 | PS22 | VNM(N) | Vu Quang NP | M | 18.286 | 105.477 | OP204537.1 | OP178808.1 | OP178688.1 |
| *Lechia squamata* | Sal-LP-0711 | PS1 | VNM(N) | Vu Quang NP | F | 18.286 | 105.477 | OP204605.1 | OP178878.1 | OP178758.1 |
| *Lechia squamata* | Sal-LP-0712 | PS1 | VNM(N) | Vu Quang NP | F | 18.286 | 105.477 | OP204607.1 | OP178880.1 | OP178760.1 |
| *Phintella* M4 | Sal-LP-0720 | PS20 | VNM(N) | Vu Quang NP | M | 18.286 | 105.477 | OP204610.1 | OP178883.1 | OP178763.1 |
| *Phintella* F5 | Sal-LP-0721 | PS13 | VNM(N) | Vu Quang NP | F | 18.286 | 105.477 | OP204513.1 | OP178783.1 | OP178663.1 |
| *Phintella aepuipeiformis* | Sal-LP-0726 | PS12 | VNM(N) | Vu Quang NP | F | 18.286 | 105.477 | LC105669.1 | OP178777.1 | OP178657.1 |
| *Phintella aequipeiformis* | Sal-LP-0727 | PS12 | VNM(N) | Vu Quang NP | F | 18.286 | 105.477 | LC105670.1 |  |  |
| *Phintella aequipeiformis* | Sal-LP-0728 | PS12 | VNM(N) | Vu Quang NP | F | 18.286 | 105.477 | LC105671.1 |  |  |
| *Phintella aepuipeiformis* | Sal-LP-0729 | PS12 | VNM(N) | Vu Quang NP | F | 18.286 | 105.477 | LC105672.1 | OP178778.1 | OP178658.1 |
| *Phintella vittata* | Sal-LP-0768 | PS17 | VNM(S) | Phu Quoc NP | M | 10.331 | 104.030 | OP204554.1 | OP178825.1 | OP178705.1 |
| *Phintella vittata* | Sal-LP-0769 | PS17 | VNM(S) | Phu Quoc NP | F | 10.331 | 104.030 | OP204555.1 | OP178826.1 | OP178706.1 |
| *Phintella* F7 | Sal-LP-0770 | PS10 | VNM(S) | Phu Quoc NP | F | 10.331 | 104.030 | OP204567.1 | OP178838.1 | OP178718.1 |
| *Phintella* M6 | Sal-LP-0775 | PS11 | VNM(S) | Phu Quoc NP | M | 10.331 | 104.030 | OP204556.1 | OP178827.1 | OP178707.1 |
| *Phintella* F6 | Sal-LP-0782 | PS11 | VNM(S) | Phu Quoc NP | F | 10.331 | 104.030 | OP204557.1 | OP178828.1 | OP178708.1 |
| *Phintella bifurcilinea* | Sal-LP-0790 | PS22 | VNM(S) | Phu Quoc NP | M | 10.331 | 104.030 | OP204519.1 | OP178790.1 | OP178670.1 |
| *Phintella* M7 | Sal-LP-0827 | PS10 | VNM(S) | Phu Quoc NP | M | 10.331 | 104.030 | OP204564.1 | OP178835.1 | OP178715.1 |
| *Phintella* F7 | Sal-LP-0828 | PS10 | VNM(S) | Phu Quoc NP | F | 10.331 | 104.030 | OP204566.1 | OP178837.1 | OP178717.1 |
| *Phintella debilis* | Sal-LP-0860 | PS22 | VNM(S) | Lo Go Xa Mat NP | M | 11.601 | 105.899 | OP204528.1 | OP178799.1 | OP178679.1 |
| *Phintella* F6 | Sal-LP-0869 | PS11 | VNM(S) | Lo Go Xa Mat NP | F | 11.601 | 105.899 | OP204558.1 | OP178829.1 | OP178709.1 |
| *Phintella* M6 | Sal-LP-0946 | PS11 | VNM(S) | Lo Go Xa Mat NP | M | 11.601 | 105.899 | OP204559.1 | OP178830.1 | OP178710.1 |
| *Phintella* F6 | Sal-LP-0967 | PS11 | VNM(S) | Lo Go Xa Mat NP | F | 11.601 | 105.899 | OP204560.1 | OP178831.1 | OP178711.1 |
| *Phintella* F6 | Sal-LP-0969 | PS11 | VNM(S) | Lo Go Xa Mat NP | F | 11.601 | 105.899 | OP204561.1 | OP178832.1 | OP178712.1 |
| *Phintella lepidus* | Sal-LP-0986 | PS6 | VNM(S) | Lo Go Xa Mat NP | F | 11.601 | 105.899 | OP204580.1 | OP178851.1 | OP178731.1 |
| *Phintella lepidus* | Sal-LP-0987 | PS6 | VNM(S) | Lo Go Xa Mat NP | M | 11.601 | 105.899 | OP204581.1 | OP178852.1 | OP178732.1 |
| *Phintella vittata* | Sal-LP-0993 | PS17 | VNM(S) | Phu Quoc NP | M | 10.331 | 104.030 | OP204543.1 | OP178814.1 | OP178694.1 |
| *Phintella lepidus* | Sal-LP-0996 | PS6 | VNM(S) | Phu Quoc NP | F | 10.331 | 104.030 | OP204579.1 | OP178850.1 | OP178730.1 |
| *Phintella* F7 | Sal-LP-0999 | PS10 | VNM(S) | Phu Quoc NP | F | 10.331 | 104.030 | OP204565.1 | OP178836.1 | OP178716.1 |
| *Phintella* F7 | Sal-LP-1000 | PS10 | VNM(S) | Phu Quoc NP | F | 10.331 | 104.030 | OP204568.1 | OP178839.1 | OP178719.1 |
| *Phintella* F7 | Sal-LP-1001 | PS10 | VNM(S) | Phu Quoc NP | F | 10.331 | 104.030 | OP204569.1 | OP178840.1 | OP178720.1 |
| *Phintella vittata* | Sal-LP-1003 | PS17 | VNM(S) | Lo Go Xa Mat NP | F | 11.601 | 105.899 | OP204542.1 | OP178813.1 | OP178693.1 |
| *Phintella vittata* | Sal-LP-1004 | PS17 | VNM(S) | Lo Go Xa Mat NP | M | 11.601 | 105.899 | OP204544.1 | OP178815.1 | OP178695.1 |
| *Phintella bifurcilinea* | Sal-LP-1065 | PS22 | VNM(N) | Ba Vi NP | M | 21.082 | 105.372 | OP204518.1 | OP178789.1 | OP178669.1 |
| *Phintella bifurcilinea* | Sal-LP-1066 | PS22 | VNM(N) | Ba Vi NP | F | 21.082 | 105.372 | OP204520.1 | OP178791.1 | OP178671.1 |
| *Phintella* F8 | Sal-LP-1068 | PS19 | VNM(N) | Ba Vi NP | F | 21.082 | 105.372 | OP204588.1 | OP178859.1 | OP178739.1 |
| *Phintella* M8 | Sal-LP-1069 | PS19 | VNM(N) | Ba Vi NP | M | 21.082 | 105.372 | OP204587.1 | OP178858.1 | OP178738.1 |
| *Phintella* M8 | Sal-LP-1070 | PS19 | VNM(N) | Ba Vi NP | M | 21.082 | 105.372 | OP204586.1 | OP178857.1 | OP178737.1 |
| *Phintella debilis* | Sal-LP-1110 | PS22 | VNM(S) | Chu Yang Sin NP | M | 12.305 | 108.388 | OP204530.1 | OP178801.1 | OP178681.1 |
| *Phintella* M9 | Sal-LP-1158 | PS8 | VNM(S) | Chu Yang Sin NP | M | 12.305 | 108.388 | OP204570.1 | OP178841.1 | OP178721.1 |
| *Phintella* M9 | Sal-LP-1159 | PS8 | VNM(S) | Chu Yang Sin NP | M | 12.305 | 108.388 | OP204571.1 | OP178842.1 | OP178722.1 |
| *Phintella* F9 | Sal-LP-1164 | PS8 | VNM(S) | Chu Yang Sin NP | F | 12.305 | 108.388 | OP204572.1 | OP178843.1 | OP178723.1 |
| *Phintella* F9 | Sal-LP-1165 | PS8 | VNM(S) | Chu Yang Sin NP | F | 12.305 | 108.388 | OP204573.1 | OP178844.1 | OP178724.1 |
| *Phintella sancha* | Sal-LP-1166 | PS7 | VNM(S) | Chu Yang Sin NP | M | 12.305 | 108.388 | OP204583.1 | OP178854.1 | OP178734.1 |
| *Phintella* M10 | Sal-LP-1203 | PS5 | VNM(C) | Yokdon NP | M | 12.934 | 107.547 | OP204582.1 | OP178853.1 | OP178733.1 |
| *Phintella* *sancha* | Sal-LP-1204 | PS7 | VNM(S) | Chu Yang Sin NP | F | 12.305 | 108.388 | OP204584.1 | OP178855.1 | OP178735.1 |
| *Phintella* F11 | Sal-LP-1205 | PS21 | VNM(C) | Yokdon NP | F | 12.934 | 107.547 | OP204539.1 | OP178810.1 | OP178690.1 |
| *Phintella monteithi* | Sal-LP-1206 | PS21 | VNM(C) | Yokdon NP | M | 12.934 | 107.547 | OP204540.1 | OP178811.1 | OP178691.1 |
| *Phintella monteithi* | Sal-LP-1207 | PS21 | VNM(C) | Yokdon NP | M | 12.934 | 107.547 | OP204541.1 | OP178812.1 | OP178692.1 |
| *Phintella debilis* | Sal-LP-1281 | PS22 | VNM(N) | Xuan Lien NR | M | 20.010 | 105.079 | OP204531.1 | OP178802.1 | OP178682.1 |
| *Phintella bifurcilinea* | Sal-LP-1294 | PS22 | VNM(N) | Xuan Lien NR | M | 20.010 | 105.079 | OP204514.1 | OP178784.1 | OP178664.1 |
| *Phintella* F8 | Sal-LP-1313 | PS19 | VNM(N) | Ben En NP | F | 19.618 | 105.529 | OP204589.1 | OP178860.1 | OP178740.1 |
| *Phintella* M11 | Sal-LP-1383 | PS18 | VNM(N) | Xuan Lien NR | M | 20.010 | 105.079 | OP204591.1 | OP178862.1 | OP178742.1 |
| *Phintella* *liui* | Sal-LP-1422 | PS9 | VNM(N) | Ben En NP | F | 19.618 | 105.529 | OP204574.1 | OP178845.1 | OP178725.1 |
| *Phintella liui* | Sal-LP-1424 | PS9 | VNM(N) | Ben En NP | M | 19.618 | 105.529 | OP204578.1 | OP178849.1 | OP178729.1 |
| *Phintella debilis* | Sal-LP-1453 | PS22 | VNM(N) | Pu Mat NP | M | 19.057 | 104.860 | OP204525.1 | OP178796.1 | OP178676.1 |
| *Phintella debilis* | Sal-LP-1454 | PS22 | VNM(N) | Pu Mat NP | F | 19.057 | 104.860 | OP204532.1 | OP178803.1 | OP178683.1 |
| *Phintella* M8 | Sal-LP-1455 | PS19 | VNM(N) | Pu Mat NP | M | 19.057 | 104.860 | OP204585.1 | OP178856.1 | OP178736.1 |
| *Phintella* F12 | Sal-LP-1492 | PS18 | VNM(N) | Pu Mat NP | F | 19.057 | 104.860 | OP204592.1 | OP178863.1 | OP178743.1 |
| *Phintella* M11 | Sal-LP-1505 | PS18 | VNM(C) | Dakrong NR | M | 16.620 | 107.011 | OP204593.1 | OP178864.1 | OP178744.1 |
| *Phintella suavis* | Sal-LP-1549 | PS17 | VNM(N) | Xuan Lien NR | M | 20.010 | 105.079 | OP204545.1 | OP178816.1 | OP178696.1 |
| *Phintella suavis* | Sal-LP-1550 | PS17 | VNM(N) | Xuan Lien NR | M | 20.010 | 105.079 | OP204550.1 | OP178821.1 | OP178701.1 |
| *Phintella vittata* | Sal-LP-1552 | PS17 | VNM(N) | Xuan Lien NR | M | 20.010 | 105.079 | OP204551.1 | OP178822.1 | OP178702.1 |
| *Phintella vittata* | Sal-LP-1557 | PS17 | VNM(N) | Xuan Lien NR | F | 20.010 | 105.079 | OP204552.1 | OP178823.1 | OP178703.1 |
| *Phintella vittata* | Sal-LP-1558 | PS17 | VNM(N) | Xuan Lien NR | F | 20.010 | 105.079 | OP204553.1 | OP178824.1 | OP178704.1 |
| *Phintella* M11 | Sal-LP-1567 | PS18 | VNM(N) | Xuan Lien NR | M | 20.010 | 105.079 | OP204594.1 | OP178865.1 | OP178745.1 |
| *Phintella* M12 | Sal-LP-1592 | PS16 | VNM(N) | Ben En NP | M | 19.618 | 105.529 | OP204595.1 | OP178866.1 | OP178746.1 |
| *Phintelloides* *pengi* | Sal-LP-1603 | PS2 | VNM(N) | Ben En NP | F | 19.618 | 105.529 | OP204603.1 | OP178876.1 | OP178756.1 |
| *Phintella* F13 | Sal-LP-1674 | PS20 | VNM(C) | K’Bang | F | 14.466 | 108.525 | OP204611.1 | OP178884.1 | OP178764.1 |
| *Phintella* F13 | Sal-LP-1675 | PS20 | VNM(C) | K’Bang | F | 14.466 | 108.525 | OP204614.1 | OP178887.1 | OP178767.1 |
| *Phintella* M6 | Sal-LP-1676 | PS11 | VNM(C) | K’Bang | M | 14.466 | 108.525 | OP204562.1 | OP178833.1 | OP178713.1 |
| *Phintella* F6 | Sal-LP-1677 | PS11 | VNM(C) | K’Bang | F | 14.466 | 108.525 | OP204563.1 | OP178834.1 | OP178714.1 |
| *Phintella* M11 | Sal-LP-1681 | PS18 | VNM(C) | K’Bang | M | 14.466 | 108.525 | OP204590.1 | OP178861.1 | OP178741.1 |
| *Phintella* M4 | Sal-LP-1714 | PS20 | VNM(C) | K’Bang | M | 14.466 | 108.525 | OP204612.1 | OP178885.1 | OP178765.1 |
| *Phintella* M4 | Sal-LP-1715 | PS20 | VNM(C) | K’Bang | M | 14.466 | 108.525 | OP204613.1 | OP178886.1 | OP178766.1 |
| *Phintella abnormis* | n/a |  | EA | East Asia |  |  |  |  |  | JN817064.1 |
| *Phintella arennicolor* | n/a |  | EA | East Asia |  |  |  |  |  | JN817065.1 |
| *Phintella cavaleriei* | n/a |  | CHN | China |  |  |  | MW540530.1 |  |  |
| *Phintella cavaleriei* | n/a |  | EA | East Asia |  |  |  |  |  | JN817067.1 |
| *Phintella argentea* | n/a |  | LKA | Sri Lanka |  |  |  | KY888763.1 |  | KY888727.1 |
| *Phintella argentea* | n/a |  | LKA | Sri Lanka |  |  |  | KY888750.1 |  | KY888722.1 |
| *Phintella jaleeli* | n/a |  | LKA | Sri Lanka |  |  |  | KY888760.1 |  | KY888748.1 |
| *Phintella jaleeli* | n/a |  | LKA | Sri Lanka |  |  |  | KY888757.1 |  | KY888745.1 |
| *Phintella linea* | n/a |  | EA | East Asia |  |  |  |  |  | JN817066.1 |
| *Phintella piatensis* | n/a |  | PHL | Philippines |  |  |  | AY297396.1 | AY296687.1 | AY297267.1 |
| *Phintella vittata* | n/a |  | IND | India |  |  |  | KT383680.1 |  |  |
| *Phintella vittata* | n/a |  | LKA | Sri Lanka |  |  |  | KY888758.1 |  | KY888746.1 |
| *Phintella vittata* | n/a |  | LKA | Sri Lanka |  |  |  | KY888751.1 |  | KY888728.1 |
| *Phintella* sp. | n/a |  | HI | Hawaii |  |  |  | AY297397.1 |  |  |
| *Phintella* sp. | n/a |  | IDN | Indonesia |  |  |  |  |  | MT596683.1 |
| *Phintelloides alborea* | n/a |  | LKA | Sri Lanka |  |  |  | KY888783.1 |  | KY888737.1 |
| *Phintelloides brunne* | n/a |  | LKA | Sri Lanka |  |  |  | KY888754.1 |  | KY888742.1 |
| *Phintelloides brunne* | n/a |  | LKA | Sri Lanka |  |  |  | KY888764.1 |  | KY888723.1 |
| *Phintelloides flavoviri* | n/a |  | LKA | Sri Lanka |  |  |  | KY888752.1 |  | KY888724.1 |
| *Phintelloides flavumi* | n/a |  | LKA | Sri Lanka |  |  |  | KY888768.1 |  | KY888730.1 |
| *Phintelloides jesudasi* | n/a |  | LKA | Sri Lanka |  |  |  | KY888753.1 |  | KY888741.1 |
| *Phintelloides alborea* | n/a |  | IND | Sri Lanka |  |  |  | KY888766.1 |  | KY888729.1 |
| *Phintelloides undulatus* | n/a |  | PAK | Pakistan |  |  |  | HQ991573.1 |  |  |
| *Phintelloides undulatus* | n/a |  | PAK | Pakistan |  |  |  | JF884322.1 |  |  |
| *Phintelloides* sp. | n/a |  | PAK | Pakistan |  |  |  | KY587572.1 |  |  |
| *Phintelloides* sp. | n/a |  | PAK | Pakistan |  |  |  | KY587573.1 |  |  |
| *Proszynskia pallidea* | n/a |  | LKA | Sri Lanka |  |  |  | KY888774.1 |  | KY888733.1 |
| *Epocilla aurantiaca* | n/a |  | LKA | Sri Lanka |  |  |  | KY888759.1 |  |  |
| *Hakka himeshimensis* | n/a |  | EA | EAST ASIA |  |  |  | JN817278.1 |  | JN817058.1 |
| *Helicius chikunii* | n/a |  | JPN | Japan |  |  |  | AB924449.1 |  |  |
| *Heliophanus cupreus* | n/a |  | DEU | Germany |  |  |  | KY270452.1 |  |  |
| *Pseudicius vulpes* | n/a |  | JPN | Japan |  |  |  | JN817279.1 |  | JN817059.1 |
| *Phintella* sp. | n/a |  | GAB | Gabon |  |  |  |  |  | KM033187.1 |
