# Supplementary material for: High species diversity of Phintella and Phintella‐like spiders (Araneae: Salticidae) in Vietnam revealed by DNA‐based species delimitation analyses
Source: Ecol Evol. 2024 Mar 12;14(3):e11144. doi: 10.1002/ece3.11144 (PMC10932738; doi:10.1002/ece3.11144)
Supplement: Supplementary file 3 — Table S2 [file ECE3-14-e11144-s002.docx]

Table S2. Summary of the species delimitation results.

| **Sample name** | **Morphospecies** | **ID code** | **COI** | **beast clade** | **bpp result** | **putative species** | **bGMYC** | **ASAP** |
| --- | --- | --- | --- | --- | --- | --- | --- | --- |
| *Proszynskia pallidea* LKA | *Proszynskia pallidea* |  | KY888774.1 | A | A | - | 1 | 30 |
| *Lechia squamata* PS1 Sal-LP-0711 VNM(N) | *Lechia squamata* | Sal-LP-0711 | OP204605.1 | A | A | PS19 | 2 | 1 |
| *Lechia squamata* PS1 Sal-LP-0568 VNM(N) | *Lechia squamata* | Sal-LP-0568 | OP204606.1 | A | A | PS19 | 2 | 1 |
| *Lechia squamata* PS1 Sal-LP-0712 VNM(N) | *Lechia squamata* | Sal-LP-0712 | OP204607.1 | A | A | PS19 | 2 | 1 |
| *Lechia* M2 PS1 Sal-LP-0636 VNM(N) | M2 | Sal-LP-0636 | OP204608.1 | A | A | PS19 | 2 | 1 |
| *Lechia* M2 PS1 Sal-LP-0637 VNM(N) | M2 | Sal-LP-0637 | OP204609.1 | A | A | PS19 | 2 | 1 |
| *Phintelloides flavoviri* LKA | *Phintelloides flavoviri* |  | KY888752.1 | B | B | - | 3 | 27 |
| *Phintelloides brunne* LKA 1 | *Phintelloides brunne* |  | KY888754 .1 | B | B | - | 4 | 26 |
| *Phintelloides brunne* LKA 2 | *Phintelloides brunne* |  | KY888764 .1 | B | B | - | 4 | 26 |
| *Phintelloides jesudasi* LKA | *Phintelloides jesudasi* |  | KY888753.1 | B | B | - | 5 | 28 |
| *Phintelloides flavumi* LKA | *Phintelloides flavumi* |  | KY888768.1 | B | B | - | 5 | 28 |
| *Phintelloides jesudasi* IND | *Phintelloides jesudasi* |  | KY888766 .1 | B | B | - | 6 | 25 |
| *Phintelloides alborea* LKA | *Phintelloides alborea* |  | KY888783.1 | B | B | - | 6 | 25 |
| *Phintelloides* *pengi* PS2 Sal-LP-0644 VNM(N) | F2 | Sal-LP-0644 | OP204601.1 | B | B | PS20 | 7 | 4 |
| *Phintelloides* *pengi* PS2 Sal-LP-0467 VNM(N) | M1 | Sal-LP-0467 | OP204602.1 | B | B | PS20 | 7 | 4 |
| *Phintelloides* *pengi* PS2 Sal-LP-1603 VNM(N) | F2 | Sal-LP-1603 | OP204603.1 | B | B | PS20 | 7 | 4 |
| *Phintelloides* *pengi* PS2 Sal-LP-0475 VNM(N) | F2 | Sal-LP-0475 | OP204604.1 | B | B | PS20 | 7 | 4 |
| *Phintelloides* F1 PS3 Sal-LP-0695 VNM(N) | F1 | Sal-LP-0695 | OP204600.1 | B | B | PS21 | 8 | 16 |
| *Phintella* sp. HI | P. sp. |  | AY297397.1 | B | B | - | 9 | 2 |
| *Phintelloides versicolor* PS4 Sal-LP-0204 VNM(N) | *Phintelloides versicolor* | Sal-LP-0204 | LC105656.1 | B | B | PS22 | 9 | 2 |
| *Phintelloides versicolor* PS4 Sal-LP-0271 VNM(N) | *Phintelloides versicolor* | Sal-LP-0271 | LC105657.1 | B | B | PS22 | 9 | 2 |
| *Phintelloides versicolor* PS4 Sal-LP-0670 VNM(N) | *Phintelloides versicolor* | Sal-LP-0670 | LC105667.1 | B | B | PS22 | 9 | 2 |
| *Phintelloides versicolor* PS4 Sal-LP-0454 VNM(N) | *Phintelloides versicolor* | Sal-LP-0454 | OP204596.1 | B | B | PS22 | 9 | 2 |
| *Phintelloides versicolor* PS4 Sal-LP-0700 VNM(N) | *Phintelloides versicolor* | Sal-LP-0700 | OP204597.1 | B | B | PS22 | 9 | 2 |
| *Phintelloides versicolor* PS4 Sal-LP-0699 VNM(N) | *Phintelloides versicolor* | Sal-LP-0699 | OP204598.1 | B | B | PS22 | 9 | 2 |
| *Phintelloides versicolor* PS4 Sal-LP-0671 VNM(N) | *Phintelloides versicolor* | Sal-LP-0671 | OP204599.1 | B | B | PS22 | 9 | 2 |
| *Phintella* M10 PS5 Sal-LP-1203 VNM(C) | M10 | Sal-LP-1203 | OP204582.1 | C | C | PS18 | 10 | 6 |
| *Phintella lepidus* PS6 Sal-LP-0996 VNM(S) | *P. lepidus* | Sal-LP-0996 | OP204579.1 | C | C | PS17 | 11 | 3 |
| *Phintella lepidus* PS6 Sal-LP-0986 VNM(S) | *P. lepidus* | Sal-LP-0986 | OP204580.1 | C | C | PS17 | 11 | 3 |
| *Phintella lepidus* PS6 Sal-LP-0987 VNM(S) | *P. lepidus* | Sal-LP-0987 | OP204581.1 | C | C | PS17 | 11 | 3 |
| *Phintelloides undulatus* PAK 1 | *Phintelloides undulatus* |  | HQ991573.1 | C | C | - | 12 | 29 |
| *Phintelloides undulatus* PAK 2 | *Phintelloides undulatus* |  | JF884322.1 | C | C | - | 12 | 29 |
| *Phintelloides* sp. PAK 1 | *Phintelloides* sp. |  | KY587572.1 | C | C | - | 12 | 29 |
| *Phintelloides* sp. PAK 2 | *Phintelloides* sp. |  | KY587573.1 | C | C | - | 12 | 29 |
| *Phintella jaleeli* LKA 2 | *P. jaleeli* |  | KY888757 .1 | D | D | - | 13 | 23 |
| *Phintella jaleeli* LKA 1 | *P. jaleeli* |  | KY888760 .1 | D | D | - | 13 | 23 |
| *Phintella sancha* PS7 Sal-LP-1166 VNM(S) | *P. sancha* | Sal-LP-1166 | OP204583.1 | D | D | PS16 | 14 | 5 |
| *Phintella* *sancha* PS7 Sal-LP-1204 VNM(S) | F10 | Sal-LP-1204 | OP204584.1 | D | D | PS16 | 14 | 5 |
| *Phintella* M9 PS8 Sal-LP-1158 VNM(S) | M9 | Sal-LP-1158 | OP204570.1 | E | E | PS1 | 15 | 9 |
| *Phintella* M9 PS8 Sal-LP-1159 VNM(S) | M9 | Sal-LP-1159 | OP204571.1 | E | E | PS1 | 15 | 9 |
| *Phintella* F9 PS8 Sal-LP-1164 VNM(S) | F9 | Sal-LP-1164 | OP204572.1 | E | E | PS1 | 15 | 9 |
| *Phintella* F9 PS8 Sal-LP-1165 VNM(S) | F9 | Sal-LP-1165 | OP204573.1 | E | E | PS1 | 15 | 9 |
| *Phintella* *liui* PS9 Sal-LP-1422 VNM(N) | F4 | Sal-LP-1422 | OP204574.1 | E | E | PS2 | 16 | 8 |
| *Phintella* *liui* PS9 Sal-LP-0590 VNM(N) | M3 | Sal-LP-0590 | OP204575.1 | E | E | PS2 | 16 | 8 |
| *Phintella* *liui* PS9 Sal-LP-0602 VNM(N) | F4 | Sal-LP-0602 | OP204576.1 | E | E | PS2 | 16 | 8 |
| *Phintella* *liui* PS9 Sal-LP-0630 VNM(N) | M3 | Sal-LP-0630 | OP204577.1 | E | E | PS2 | 16 | 8 |
| *Phintella* *liui* PS9 Sal-LP-1424 VNM(N) | M3 | Sal-LP-1424 | OP204578.1 | E | E | PS2 | 16 | 8 |
| *Phintella* M7 PS10 Sal-LP-0827 VNM(S) | M7 | Sal-LP-0827 | OP204564.1 | F | F | PS4 | 17 | 10 |
| *Phintella* F7 PS10 Sal-LP-0999 VNM(S) | F7 | Sal-LP-0999 | OP204565.1 | F | F | PS4 | 17 | 10 |
| *Phintella* F7 PS10 Sal-LP-0828 VNM(S) | F7 | Sal-LP-0828 | OP204566.1 | F | F | PS4 | 17 | 10 |
| *Phintella* F7 PS10 Sal-LP-0770 VNM(S) | F7 | Sal-LP-0770 | OP204567.1 | F | F | PS4 | 17 | 10 |
| *Phintella* F7 PS10 Sal-LP-1000 VNM(S) | F7 | Sal-LP-1000 | OP204568.1 | F | F | PS4 | 17 | 10 |
| *Phintella* F7 PS10 Sal-LP-1001 VNM(S) | F7 | Sal-LP-1001 | OP204569.1 | F | F | PS4 | 17 | 10 |
| *Phintella* M6 PS11 Sal-LP-0775 VNM(S) | M6 | Sal-LP-0775 | OP204556.1 | F | F | PS3 | 18 | 11 |
| *Phintella* F6 PS11 Sal-LP-0782 VNM(S) | F6 | Sal-LP-0782 | OP204557.1 | F | F | PS3 | 18 | 11 |
| *Phintella* F6 PS11 Sal-LP-0869 VNM(S) | F6 | Sal-LP-0869 | OP204558.1 | F | F | PS3 | 18 | 11 |
| *Phintella* M6 PS11 Sal-LP-0946 VNM(S) | M6 | Sal-LP-0946 | OP204559.1 | F | F | PS3 | 18 | 11 |
| *Phintella* F6 PS11 Sal-LP-0967 VNM(S) | F6 | Sal-LP-0967 | OP204560.1 | F | F | PS3 | 18 | 11 |
| *Phintella* F6 PS11 Sal-LP-0969 VNM(S) | F6 | Sal-LP-0969 | OP204561.1 | F | F | PS3 | 18 | 11 |
| *Phintella* M6 PS11 Sal-LP-1676 VNM(C) | M6 | Sal-LP-1676 | OP204562.1 | F | F | PS3 | 18 | 11 |
| *Phintella* F6 PS11 Sal-LP-1677 VNM(C) | F6 | Sal-LP-1677 | OP204563.1 | F | F | PS3 | 18 | 11 |
| *Phintella aepuipeiformis* PS12 Sal-LP-0329 VNM(N) | *P. aepuipeiformis* | Sal-LP-0329 | LC105658.1 | G | G | PS10 | 22 | 22 |
| *Phintella aepuipeiformis* PS12 Sal-LP-0490 VNM(N) | *P. aepuipeiformis* | Sal-LP-0490 | LC105659.1 | G | G | PS10 | 22 | 22 |
| *Phintella aepuipeiformis* PS12 Sal-LP-0491 VNM(N) | *P. aepuipeiformis* | Sal-LP-0491 | LC105660.1 | G | G | PS10 | 22 | 22 |
| *Phintella aepuipeiformis* PS12 Sal-LP-0531 VNM(N) | *P. aepuipeiformis* | Sal-LP-0531 | LC105661.1 | G | G | PS10 | 22 | 22 |
| *Phintella aequipeiformis* PS12 Sal-LP-0586 VNM(N) | *P. aequipeiformis* | Sal-LP-0586 | LC105662.1 | G | G | PS10 | 22 | 22 |
| *Phintella aequipeiformis* PS12 Sal-LP-0587 VNM(N) | *P. aequipeiformis* | Sal-LP-0587 | LC105663.1 | G | G | - | 22 | 22 |
| *Phintella aepuipeiformis* PS12 Sal-LP-0589 VNM(N) | *P. aepuipeiformis* | Sal-LP-0589 | LC105665.1 | G | G | PS10 | 22 | 22 |
| *Phintella aepuipeiformis* PS12 Sal-LP-0622 VNM(N) | *P. aepuipeiformis* | Sal-LP-0622 | LC105666.1 | G | G | PS10 | 22 | 22 |
| *Phintella aepuipeiformis* PS12 Sal-LP-0726 VNM(N) | *P. aepuipeiformis* | Sal-LP-0726 | LC105669.1 | G | G | PS10 | 22 | 22 |
| *Phintella aequipeiformis* PS12 Sal-LP-0727 VNM(N) | *P. aequipeiformis* | Sal-LP-0727 | LC105670.1 | G | G | PS10 | 22 | 22 |
| *Phintella aequipeiformis* PS12 Sal-LP-0728 VNM(N) | *P. aequipeiformis* | Sal-LP-0728 | LC105671.1 | G | G | PS10 | 22 | 22 |
| *Phintella aepuipeiformis* PS12 Sal-LP-0729 VNM(N) | *P. aepuipeiformis* | Sal-LP-0729 | LC105672.1 | G | G | PS10 | 22 | 22 |
| *Phintella* M5 PS13 Sal-LP-0632 VNM(N) | M5 | Sal-LP-0632 | OP204512.1 | H | H | PS15 | 23 | 17 |
| *Phintella* F5 PS13 Sal-LP-0721 VNM(N) | F5 | Sal-LP-0721 | OP204513.1 | H | H | PS15 | 23 | 17 |
| *Phintella* F3 PS14 Sal-LP-0160 VNM(N) | F3 | Sal-LP-0160 | OP204510.1 | H | H | PS13 | 24 | 14 |
| *Phintella cavaleriei* CHN | *P. cavaleriei* |  | MW540530.1 | H | H | - | 25 | 7 |
| *Phintella cavaleriei* PS15 Sal-LP-0250 VNM(N) | *P. cavaleriei* | Sal-LP-0250 | OP204511.1 | H | H | PS14 | 25 | 7 |
| *Phintella* M12 PS16 Sal-LP-1592 VNM(N) | M12 | Sal-LP-1592 | OP204595.1 | H | H | PS11 | 26 | 13 |
| *Phintella argentea* LKA 2 | *P. argentea* |  | KY888750 .1 | H | H | - | 27 | 15 |
| *Phintella argentea* LKA 1 | *P. argentea* |  | KY888763 .1 | H | H | - | 27 | 15 |
| *Phintella vittata* IND | *P. vittata* |  | KT383680.1 | H | H | - | 28 | 15 |
| *Phintella vittata* LKA 2 | *P. vittata* |  | KY888751.1 | H | H | - | 29 | 15 |
| *Phintella vittata* LKA 1 | *P. vittata* |  | KY888758 .1 | H | H | - | 29 | 15 |
| *Phintella vittata* PS17 Sal-LP-1003 VNM(S) | *P. vittata* | Sal-LP-1003 | OP204542.1 | H | H | PS12 | 30 | 15 |
| *Phintella vittata* PS17 Sal-LP-0993 VNM(S) | *P. vittata* | Sal-LP-0993 | OP204543.1 | H | H | PS12 | 30 | 15 |
| *Phintella vittata* PS17 Sal-LP-1004 VNM(S) | *P. vittata* | Sal-LP-1004 | OP204544.1 | H | H | PS12 | 30 | 15 |
| *Phintella suavis* PS17 Sal-LP-1549 VNM(N) | *P. suavis* | Sal-LP-1549 | OP204545.1 | H | H | PS12 | 30 | 15 |
| *Phintella vittata* PS17 Sal-LP-0476 VNM(N) | *P. vittata* | Sal-LP-0476 | OP204546.1 | H | H | PS12 | 30 | 15 |
| *Phintella vittata* PS17 Sal-LP-0477 VNM(N) | *P. vittata* | Sal-LP-0477 | OP204547.1 | H | H | PS12 | 30 | 15 |
| *Phintella vittata* PS17 Sal-LP-0674 VNM(N) | *P. vittata* | Sal-LP-0674 | OP204548.1 | H | H | PS12 | 30 | 15 |
| *Phintella suavis* PS17 Sal-LP-0675 VNM(N) | *P. suavis* | Sal-LP-0675 | OP204549.1 | H | H | PS12 | 30 | 15 |
| *Phintella suavis* PS17 Sal-LP-1550 VNM(N) | *P. suavis* | Sal-LP-1550 | OP204550.1 | H | H | PS12 | 30 | 15 |
| *Phintella vittata* PS17 Sal-LP-1552 VNM(N) | *P. vittata* | Sal-LP-1552 | OP204551.1 | H | H | PS12 | 30 | 15 |
| *Phintella vittata* PS17 Sal-LP-1557 VNM(N) | *P. vittata* | Sal-LP-1557 | OP204552.1 | H | H | PS12 | 30 | 15 |
| *Phintella vittata* PS17 Sal-LP-1558 VNM(N) | *P. vittata* | Sal-LP-1558 | OP204553.1 | H | H | PS12 | 30 | 15 |
| *Phintella vittata* PS17 Sal-LP-0768 VNM(S) | *P. vittata* | Sal-LP-0768 | OP204554.1 | H | H | PS12 | 30 | 15 |
| *Phintella vittata* PS17 Sal-LP-0769 VNM(S) | *P. vittata* | Sal-LP-0769 | OP204555.1 | H | H | PS12 | 30 | 15 |
| *Phintella piatensis* PHL | *P. piatensis* |  | AY297396.1 | I | I | - | 31 | 24 |
| *Phintella* M11 PS18 Sal-LP-1681 VNM(C) | M11 | Sal-LP-1681 | OP204590.1 | I | I | PS8 | 32 | 20 |
| *Phintella* M11 PS18 Sal-LP-1383 VNM(N) | M11 | Sal-LP-1383 | OP204591.1 | I | I | PS8 | 32 | 20 |
| *Phintella* F12 PS18 Sal-LP-1492 VNM(N) | F12 | Sal-LP-1492 | OP204592.1 | I | I | PS8 | 32 | 20 |
| *Phintella* M11 PS18 Sal-LP-1505 VNM(C) | M11 | Sal-LP-1505 | OP204593.1 | I | I | PS8 | 32 | 20 |
| *Phintella* M11 PS18 Sal-LP-1567 VNM(N) | M11 | Sal-LP-1567 | OP204594.1 | I | I | PS8 | 32 | 20 |
| *Phintella* M8 PS19 Sal-LP-1455 VNM(N) | M8 | Sal-LP-1455 | OP204585.1 | I | I | PS7 | 33 | 21 |
| *Phintella* M8 PS19 Sal-LP-1070 VNM(N) | M8 | Sal-LP-1070 | OP204586.1 | I | I | PS7 | 33 | 21 |
| *Phintella* M8 PS19 Sal-LP-1069 VNM(N) | M8 | Sal-LP-1069 | OP204587.1 | I | I | PS7 | 33 | 21 |
| *Phintella* F8 PS19 Sal-LP-1068 VNM(N) | F8 | Sal-LP-1068 | OP204588.1 | I | I | PS7 | 33 | 21 |
| *Phintella* F8 PS19 Sal-LP-1313 VNM(N) | F8 | Sal-LP-1313 | OP204589.1 | I | I | PS7 | 33 | 21 |
| *Phintella* M4 PS20 Sal-LP-0720 VNM(N) | M4 | Sal-LP-0720 | OP204610.1 | I | I | PS9 | 35 | 12 |
| *Phintella* F13 PS20 Sal-LP-1674 VNM(C) | F13 | Sal-LP-1674 | OP204611.1 | I | I | PS9 | 35 | 12 |
| *Phintella* M4 PS20 Sal-LP-1714 VNM(C) | M4 | Sal-LP-1714 | OP204612.1 | I | I | PS9 | 35 | 12 |
| *Phintella* M4 PS20 Sal-LP-1715 VNM(C) | M4 | Sal-LP-1715 | OP204613.1 | I | I | - | 35 | 12 |
| *Phintella* F13 PS20 Sal-LP-1675 VNM(C) | F13 | Sal-LP-1675 | OP204614.1 | I | I | PS9 | 35 | 12 |
| *Phintella* F11 PS21 Sal-LP-1205 VNM(C) | F11 | Sal-LP-1205 | OP204539.1 | I | I | PS6 | 36 | 18 |
| *Phintella monteithi* PS21 Sal-LP-1206 VNM(C) | *P. monteithi* | Sal-LP-1206 | OP204540.1 | I | I | PS6 | 36 | 18 |
| *Phintella monteithi* PS21 Sal-LP-1207 VNM(C) | *P. monteithi* | Sal-LP-1207 | OP204541.1 | I | I | PS6 | 36 | 18 |
| *Phintella bifurcilinea* PS22 Sal-LP-0148 VNM(N) | *P. bifurcilinea* | Sal-LP-0148 | LC105655.1 | I | I | PS5 | 37 | 19 |
| *Phintella bifurcilinea* PS22 Sal-LP-0681 VNM(N) | *P. bifurcilinea* | Sal-LP-0681 | LC105668.1 | I | I | PS5 | 37 | 19 |
| *Phintella bifurcilinea* PS22 Sal-LP-1294 VNM(N) | *P. bifurcilinea* | Sal-LP-1294 | OP204514.1 | I | I | PS5 | 37 | 19 |
| *Phintella bifurcilinea* PS22 Sal-LP-0679 VNM(N) | *P. bifurcilinea* | Sal-LP-0679 | OP204515.1 | I | I | PS5 | 37 | 19 |
| *Phintella bifurcilinea* PS22 Sal-LP-0680 VNM(N) | *P. bifurcilinea* | Sal-LP-0680 | OP204516.1 | I | I | PS5 | 37 | 19 |
| *Phintella bifurcilinea* PS22 Sal-LP-0689 VNM(N) | *P. bifurcilinea* | Sal-LP-0689 | OP204517.1 | I | I | PS5 | 37 | 19 |
| *Phintella bifurcilinea* PS22 Sal-LP-1065 VNM(N) | *P. bifurcilinea* | Sal-LP-1065 | OP204518.1 | I | I | PS5 | 37 | 19 |
| *Phintella bifurcilinea* PS22 Sal-LP-0790 VNM(S) | *P. bifurcilinea* | Sal-LP-0790 | OP204519.1 | I | I | PS5 | 37 | 19 |
| *Phintella bifurcilinea* PS22 Sal-LP-1066 VNM(N) | *P. bifurcilinea* | Sal-LP-1066 | OP204520.1 | I | I | PS5 | 37 | 19 |
| *Phintella bifurcilinea* PS22 Sal-LP-0558 VNM(N) | *P. bifurcilinea* | Sal-LP-0558 | OP204522.1 | I | I | PS5 | 37 | 19 |
| *Phintella bifurcilinea* PS22 Sal-LP-0527 VNM(N) | *P. bifurcilinea* | Sal-LP-0527 | OP204523.1 | I | I | PS5 | 37 | 19 |
| *Phintella debilis* PS22 Sal-LP-0555 VNM(N) | *P. debilis* | Sal-LP-0555 | OP204524.1 | I | I | PS5 | 37 | 19 |
| *Phintella debilis* PS22 Sal-LP-1453 VNM(N) | *P. debilis* | Sal-LP-1453 | OP204525.1 | I | I | PS5 | 37 | 19 |
| *Phintella debilis* PS22 Sal-LP-0526 VNM(N) | *P. debilis* | Sal-LP-0526 | OP204526.1 | I | I | PS5 | 37 | 19 |
| *Phintella debilis* PS22 Sal-LP-0556 VNM(N) | *P. debilis* | Sal-LP-0556 | OP204527.1 | I | I | PS5 | 37 | 19 |
| *Phintella debilis* PS22 Sal-LP-0860 VNM(S) | *P. debilis* | Sal-LP-0860 | OP204528.1 | I | I | PS5 | 37 | 19 |
| *Phintella debilis* PS22 Sal-LP-0628 VNM(N) | *P. debilis* | Sal-LP-0628 | OP204529.1 | I | I | PS5 | 37 | 19 |
| *Phintella debilis* PS22 Sal-LP-1110 VNM(S) | *P. debilis* | Sal-LP-1110 | OP204530.1 | I | I | PS5 | 37 | 19 |
| *Phintella debilis* PS22 Sal-LP-1281 VNM(N) | *P. debilis* | Sal-LP-1281 | OP204531.1 | I | I | PS5 | 37 | 19 |
| *Phintella debilis* PS22 Sal-LP-1454 VNM(N) | *P. debilis* | Sal-LP-1454 | OP204532.1 | I | I | PS5 | 37 | 19 |
| *Phintella debilis* PS22 Sal-LP-0691 VNM(N) | *P. debilis* | Sal-LP-0691 | OP204533.1 | I | I | PS5 | 37 | 19 |
| *Phintella debilis* PS22 Sal-LP-0692 VNM(N) | *P. debilis* | Sal-LP-0692 | OP204534.1 | I | I | PS5 | 37 | 19 |
| *Phintella debilis* PS22 Sal-LP-0702 VNM(N) | *P. debilis* | Sal-LP-0702 | OP204535.1 | I | I | PS5 | 37 | 19 |
| *Phintella debilis* PS22 Sal-LP-0693 VNM(N) | *P. debilis* | Sal-LP-0693 | OP204536.1 | I | I | PS5 | 37 | 19 |
| *Phintella debilis* PS22 Sal-LP-0706 VNM(N) | *P. debilis* | Sal-LP-0706 | OP204537.1 | I | I | PS5 | 37 | 19 |
| *Phintella debilis* PS22 Sal-LP-0694 VNM(N) | *P. debilis* | Sal-LP-0694 | OP204538.1 | I | I | PS5 | 37 | 19 |
