# Supplementary material for: High species diversity of Phintella and Phintella‐like spiders (Araneae: Salticidae) in Vietnam revealed by DNA‐based species delimitation analyses
Source: Ecol Evol. 2024 Mar 12;14(3):e11144. doi: 10.1002/ece3.11144 (PMC10932738; doi:10.1002/ece3.11144)
Supplement: Supplementary file 4 — Table S3 [file ECE3-14-e11144-s004.docx]

Table S3. Species used in RASP analysis.

| **Tip Name** | **country** | **RASP State** | **bGMYC** |
| --- | --- | --- | --- |
| *Phintella abnormis* EA | unknown(EAST ASIA) | D | 21 |
| *Phintella aepuipeiformis* PS12 Sal-LP-0726 VNM(N) | Viet Nam | A | 22 |
| *Phintella arennicolor* EA | unknown(EAST ASIA) | D | 34 |
| *Phintella argentea* LKA 1 | Sri Lanka | E | 29 |
| *Phintella bifurcilinea* PS22 Sal-LP-0790 VNM(S) | Viet Nam | AC | 37 |
| *Phintella cavaleriei* PS15 Sal-LP-0250 VNM(N) | Viet Nam | AD | 25 |
| *Phintella cavaleriei* EA | unknown(EAST ASIA) | D | 20 |
| *Phintella jaleeli* LKA 2 | Sri Lanka | E | 13 |
| *Phintella lepidus* PS6 Sal-LP-0996 VNM(S) | Viet Nam | C | 11 |
| *Phintella monteithi* PS21 Sal-LP-1206 VNM(C) | Viet Nam | B | 36 |
| *Phintella piatensis* PHL | Philippines | F | 31 |
| *Phintella sancha* PS7 Sal-LP-1166 VNM(S) | Viet Nam | C | 14 |
| *Phintella* M6 PS11 Sal-LP-1676 VNM(C) | Viet Nam | BC | 18 |
| *Phintella* F7 PS10 Sal-LP-1001 VNM(S) | Viet Nam | C | 17 |
| *Phintella* M5 PS13 Sal-LP-0632 VNM(N) | Viet Nam | A | 30 |
| *Phintella* M12 PS16 Sal-LP-1592 VNM(N) | Viet Nam | A | 30 |
| *Phintella vittata* LKA 1 | Sri Lanka | EF | 30 |
| *Phintelloides* F1 PS3 Sal-LP-0695 VNM(N) | Viet Nam | A | 8 |
| *Phintella* F3 PS14 Sal-LP-0160 VNM(N) | Viet Nam | A | 24 |
| *Phintella* F8 PS19 Sal-LP-1068 VNM(N) | Viet Nam | A | 32 |
| *Phintella* M9 PS8 Sal-LP-1159 VNM(S) | Viet Nam | C | 15 |
| *Phintella* F13 PS20 Sal-LP-1675 VNM(C) | Viet Nam | AB | 33 |
| *Phintelloides pengi* PS2 Sal-LP-1603 VNM(N) | Viet Nam | A | 7 |
| *Phintella liui* PS9 Sal-LP-0590 VNM(N) | Viet Nam | A | 16 |
| *Phintella* M11 PS18 Sal-LP-1383 VNM(N) | Viet Nam | AB | 35 |
| *Phintella* M10 PS5 Sal-LP-1203 VNM(C) | Viet Nam | B | 10 |
| *Phintelloides versicolor* PS4 Sal-LP-0454 VNM(N) | Viet Nam | AG | 9 |
| *Phintella* sp. GAB | Gabon | H | 19 |
| *Phintella vittata* PS17 Sal-LP-0768 VNM(S) | Viet Nam | AC | 30 |
| *Phintella vittata* IND | India | E | 26 |
| *Phintelloides alborea* LKA 1 | Sri Lanka | E | 6 |
| *Phintelloides brunne* LKA 2 | Sri Lanka | E | 4 |
| *Phintelloides flavoviri* LKA | Sri Lanka | E | 3 |
| *Phintelloides jesudasi* LKA | Sri Lanka | E | 5 |
| *Phintelloides* sp. PAK 2 | Pakistan | E | 12 |
| *Proszynskia pallidea* LKA | Sri Lanka | E | 1 |
| *Lechia squamata* PS1 Sal-LP-0568 VNM(N) | Viet Nam | A | 2 |
